# A Tumor Environment‐Activated Photosensitized Biomimetic Nanoplatform for Precise Photodynamic Immunotherapy of Colon Cancer

**DOI:** 10.1002/advs.202402465

**Published:** 2024-05-10

**Authors:** Mengmeng Xiong, Ying Zhang, Huan Zhang, Qiaoqiao Shao, Qifan Hu, Junjie Ma, Yiqun Wan, Lan Guo, Xin Wan, Haitao Sun, Zhongyi Yuan, Hao Wan

**Affiliations:** ^1^ School of Chemistry and Chemical Engineering Nanchang University Nanchang 330031 P. R. China; ^2^ State Key Laboratory of Food Science and Resources Nanchang University Nanchang 330047 P. R. China; ^3^ State Key Laboratory of Precision Spectroscopy School of Physics and Electronic Science East China Normal University Shanghai 200241 P. R. China; ^4^ Postdoctoral Innovation Practice Base The First Affiliated Hospital Jiangxi Medical College Nanchang University Nanchang 330006 P. R. China

**Keywords:** anti‐tumor immune response, biomimetic nanoplatform, evasion of immune surveillance, tumor environment‐activated photodynamic therapy, tumor targeting

## Abstract

Aggressive nature of colon cancer and current imprecise therapeutic scenarios simulate the development of precise and effective treatment strategies. To achieve this, a tumor environment‐activated photosensitized biomimetic nanoplatform (PEG_2000_‐SiNcTI‐Ph/CpG‐ZIF‐8@CM) is fabricated by encapsulating metal‐organic framework loaded with developed photosensitizer PEG_2000_‐SiNcTI‐Ph and immunoadjuvant CpG oligodeoxynucleotide within fusion cell membrane expressing programmed death protein 1 (PD‐1) and cluster of differentiation 47 (CD47). By stumbling across, systematic evaluation, and deciphering with quantum chemical calculations, a unique attribute of tumor environment (low pH plus high concentrations of adenosine 5′‐triphosphate (ATP))‐activated photodynamic effect sensitized by long‐wavelength photons is validated for PEG_2000_‐SiNcTI‐Ph/CpG‐ZIF‐8@CM, advancing the precision of cancer therapy. Moreover, PEG_2000_‐SiNcTI‐Ph/CpG‐ZIF‐8@CM evades immune surveillance to target CT26 colon tumors in mice mediated by CD47/signal regulatory proteins α (SIRPα) interaction and PD‐1/programmed death ligand 1 (PD‐L1) interaction, respectively. Tumor environment‐activated photodynamic therapy realized by PEG_2000_‐SiNcTI‐Ph/CpG‐ZIF‐8@CM induces immunogenic cell death (ICD) to elicit anti‐tumor immune response, which is empowered by enhanced dendritic cells (DC) uptake of CpG and PD‐L1 blockade contributed by the nanoplatform. The photodynamic immunotherapy efficiently combats primary and distant CT26 tumors, and additionally generates immune memory to inhibit tumor recurrence and metastasis. The nanoplatform developed here provides insights for the development of precise cancer therapeutic strategies.

## Graphical abstract

A photosensitized biomimetic nanoplatform (PEG_2000_‐SiNcTI‐Ph/CpG‐ZIF‐8@CM) activated by tumor environment is designed for precise therapy of colon cancer. The ability of developed molecular photosensitizer PEG_2000_‐SiNcTI‐Ph to generate ROS sensitized by long‐wavelength photons is dormant after being loaded into ZIF‐8. Responding to tumor environment (low pH plus high ATP concentrations), PEG_2000_‐SiNcTI‐Ph/CpG‐ZIF‐8@CM is degraded to restore ROS generating capacity sensitized by long‐wavelength photons.

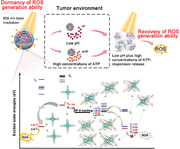

## 1 Introduction

Colon cancer is a malignant tumor with high incidence and mortality worldwide, which seriously affects human health.^[^ ^1^ ^]^ Different therapeutic modalities, such as surgery, radiotherapy, chemotherapy, etc., have been exploited to elicit a certain anti‐tumor effect. However, limitations, including but not limited to lack of specificity toward tumor tissues, off targeting, high recurrence and metastasis rates, and damage to the immune system, have stimulated to explore alternative strategies.^[^ ^2^, ^3^, ^4^ ^]^ With the rapid development of nanomedicines and nanomaterials, more precise and effective modalities are promising to be developed for treatment of colon cancer.^[^ ^5^, ^6^, ^7^ ^]^

Photodynamic therapy (PDT) has been proved to be a non‐invasive therapeutic modality with high efficacy for various tumors in clinical applications.^[^ ^8^ ^]^ During the PDT process, photosensitizers (PSs) generate cytotoxic reactive oxygen species (ROS) under laser irradiation to directly induce tumor cell apoptosis or necrosis.^[^ ^9^ ^]^ Regretfully, most of PDT developed so far realized the tumor‐specific treatment only by elaborately manipulating the direction of laser irradiation toward the tumor, proposing a high demand for the focusing of laser irradiation and the necessity to pinpoint the exact location of tumors in advance, otherwise damages to normal tissues were inevitable due to the uncontrollable diffusion of PSs, severely hindering the clinical applications of PDT. Therefore, tumor environment‐activated PDT, of which the photodynamic therapeutic process can only be initiated under tumor‐specific conditions, appears to be a more reliable and robust scenario to advance the accuracy of PDT, matching the concept of precise cancer therapy. As far as we know, now there are some nanoplatforms reported could achieve tumor environment‐activated PDT.^[^ ^10^, ^11^ ^]^ However, a few characteristics not suitable for robust in vivo applications in these work essentially need to be improved. For one thing, the utilization of PSs sensitive to short‐wavelength visible photons (such as, chlorin e6, 635 nm; methylene blue, 635 nm)^[^ ^12^, ^13^ ^]^ would result in the limited tissue penetration depth due to strong photon scattering in tissues.^[^ ^14^ ^]^ For another thing, the implementation of tumor environment‐activated PDT in some nanoplatform required elaborate control of the molar ratio and distance between PSs and the quencher, greatly limiting the robust application.^[^ ^15^ ^]^ In this regard, it is imperative to develop an easily‐handling tumor environment‐activated PDT platform with long‐wavelength photon sensitization (e.g., 750–850 nm, a window more transparent to tissues^[^ ^16^ ^]^).

Besides the direct damage, PDT‐caused killing of tumor cells can further release tumor‐associated antigens (TAAs),^[^ ^17^ ^]^ inducing immunogenic cell death (ICD) to trigger anti‐tumor immune response, which is expected to boost the cancer therapy. Recently, significant efforts have been devoted to introducing immune adjuvants to amplify anti‐tumor immune response.^[^ ^18^, ^19^ ^]^ CpG oligodeoxynucleotide is a synthetic short DNA fragment that can trigger innate immunity and induce cytokine secretion by activating dendritic cells (DCs).^[^ ^20^, ^21^ ^]^ Nevertheless, the inferior pharmacokinetics of CpG (e.g., non‐specific diffusion and low cell uptake due to the anionic property) plus the easy degradation and clearance within blood attenuated the function of CpG as an immune adjuvant to the greatest extent. Moreover, anti‐tumor immune response is seriously weakened by the immunosuppressive mechanism within the tumor.^[^ ^22^ ^]^ For instance, programmed death‐ligand 1 (PD‐L1), an immune checkpoint expressed on tumor cells, especially for colon tumor cells,^[^ ^23^ ^]^ can bind to programmed death protein 1 (PD‐1) on T lymphocytes and then shut down the immune recognition. Though approved by Food and Drug Administration (FDA) for the blockade of PD‐L1 to advance immune response, the clinical application of the anti‐PD‐L1 antibody is impeded by the high cost and inferior pharmacokinetics (e.g., clearance by the innate immune system).^[^ ^24^ ^]^ Therefore, a platform, which can improve in vivo pharmacokinetics of economical alternatives for immune checkpoint PD‐L1 blockade and CpG, appears to be the best scenario to empower photodynamic immunotherapy of colon cancer.

Zeolitic imidazolate framework‐8 (ZIF‐8), a kind of metal‐organic framework, has been gradually developed into the most representative delivery nanocarrier owing to its unique characteristics of superior biocompatibility,^[^ ^21^ ^]^ selective biodegradability toward tumor environment (i.e., high concentrations of adenosine 5′‐triphosphate (ATP)^[^ ^25^, ^26^ ^]^ plus low pH^[^ ^27^ ^]^), and versatile surface modification.^[^ ^28^, ^29^ ^]^ Although ZIF‐8 can protect the loaded agents from physiological assaults^[^ ^30^ ^]^ (e.g., endogenous enzymes and high concentration of salts) and enhance the cell uptake of loaded agents,^[^ ^31^ ^]^ as exogenous NPs, they are easily surveilled by the body's immune system for elimination, thus limiting their drug delivery efficiency in vivo. Inspired by natural biological systems, cell membrane‐based biomimetic delivery systems have attracted widespread attentions, due to the inheritance of functionalities from parental cells.^[^ ^27^, ^32^, ^33^, ^34^, ^35^, ^36^ ^]^ Specifically, the self‐recognition protein cluster of differentiation 47 (CD47) expressed on the surface of red blood cell (RBC) membrane (RM) can activate the CD47/signal regulatory proteins α (SIRPα) pathway to escape macrophage clearance to realize long blood circulation, enlightening us that this property can be utilized to improve the in vivo pharmacokinetics of administrated agents.^[^ ^37^ ^]^ As such, for the purpose of efficient tumor treatment based on the photodynamic immunotherapy, the cell membrane‐based biomimetic concept also motivates us to explore a reliable and easily‐obtained source of membrane mimicking T cell membrane, which demonstrates to be an economical alternative of the PD‐L1 antibody by simple culturing and self‐proliferation of parental cells,^[^ ^38^ ^]^ to fabricate the delivery nanoplatform. This would endow the delivery nanoplatform not only with the capacity of restoration of anti‐tumor immune response, but also with the tumor targeting ability, both of which would advance the anti‐tumor effect.

**Scheme 1:**
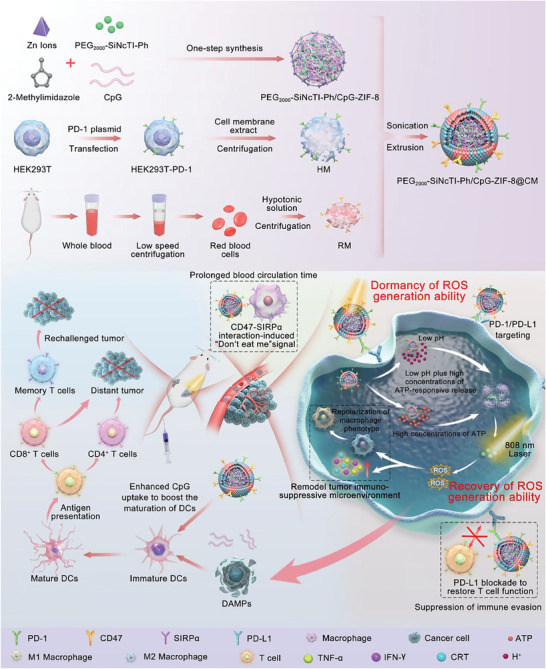
Schematic illustration of PEG_2000_‐SiNcTI‐Ph/CpG‐ZIF‐8@CM for precise photodynamic immunotherapy of CT26 colon cancer. PEG_2000_‐SiNcTI‐Ph/CpG‐ZIF‐8@CM was synthesized by embedding our developed molecular PS PEG_2000_‐SiNcTI‐Ph and immunoadjuvant CpG into the porous organic framework ZIF‐8 and finally encapsulating within the CM consisting of HM and RM. When the nanocomposite was intravenously injected into CT26 tumor‐bearing mice, they would target the tumor due to CD47/SIRPα interaction‐mediated prolonged circulation and PD‐1/PD‐L1 interaction‐mediated endocytosis. Afterward, the photodynamic effect of PEG_2000_‐SiNcTI‐Ph was restored in acidic and ATP‐rich tumor environment. Under 808 nm laser irradiation, PEG_2000_‐SiNcTI‐Ph generated sufficient ROS to kill tumor cells and released TAAs to stimulate immune response, which would be empowered by immune checkpoint PD‐L1 blockade and CpG from PEG_2000_‐SiNcTI‐Ph/CpG‐ZIF‐8@CM. As a result, PEG_2000_‐SiNcTI‐Ph/CpG‐ZIF‐8@CM realized precise and efficient photodynamic immunotherapy to combat CT26 tumors and prevent their recurrence and metastasis.

In this work, a tumor environment‐activated photosensitized biomimetic nanoplatform (PEG_2000_‐SiNcTI‐Ph/CpG‐ZIF‐8@CM) was fabricated through co‐loading our developed molecular PS PEG_2000_‐SiNcTI‐Ph and immunoadjuvant CpG into ZIF‐8 NPs, followed by encapsulation within the fusion cell membranes (CM) consisting of cell membrane overexpressing PD‐1 (HM) obtained from genetically engineered HEK293T cells (HEK293T‐PD‐1 cells) and RM,^[^ ^39^ ^]^ which could exert precise and effective photodynamic immunotherapy of CT26 colon cancer in mice sensitized by long‐wavelength photons. Camouflaged by CM, PEG_2000_‐SiNcTI‐Ph/CpG‐ZIF‐8@CM could evade immune surveillance by activating the CD47/SIRPα pathway to target CT26 tumor cells mediated by PD‐1/PD‐L1 interaction. Dormancy of the photodynamic effect of PEG_2000_‐SiNcTI‐Ph can be reversed to activate PDT once the nanoplatform was specifically degraded in response to tumor environment (i.e., low pH plus high concentrations of ATP). The PDT‐triggered ICD of CT26 tumor cells combined with enhanced DC uptake of CpG to promote maturation of DCs contributed by the nanoplatform delivery elicited anti‐tumor immune response, which was empowered by immune checkpoint PD‐L1 blockade by CM. As a result, PEG_2000_‐SiNcTI‐Ph/CpG‐ZIF‐8@CM realized precise and efficient photodynamic immunotherapy to combat CT26 tumors and prevent their recurrence and metastasis (**Scheme** **1**). The strategy we developed here would advance the development of precise cancer therapy.

## 2 Results and Discussion

### 2.1 Preparation and Characterization of PEG_2000_‐SiNcTI‐Ph/CpG‐ZIF‐8@CM

**Figure 1:**
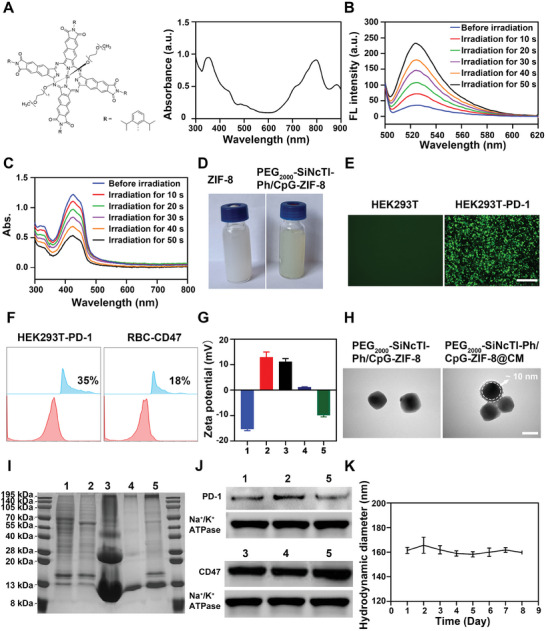
Characterization of PEG_2000_‐SiNcTI‐Ph/CpG‐ZIF‐8@CM. A) Structural formula (left picture) and UV–vis absorption spectrum (right picture) of PEG_2000_‐SiNcTI‐Ph. B,C) ROS generation ability of PEG_2000_‐SiNcTI‐Ph under 808 nm laser irradiation measured by DCFH‐DA (B) and DPBF (C). D) Digital photographs of ZIF‐8 and PEG_2000_‐SiNcTI‐Ph/CpG‐ZIF‐8 in PBS. E) Fluorescence images of HEK293T and HEK293T‐PD‐1 cells. Scale bar, 275 µm. F) Flow cytometric analysis of PD‐1 on HEK293T‐PD‐1 cells and CD47 on RBC. G) The zeta potential of different materials. (1: CM, 2: ZIF‐8, 3: PEG_2000_‐SiNcTI‐Ph‐ZIF‐8, 4: PEG_2000_‐SiNcTI‐Ph/CpG‐ZIF‐8, 5: PEG_2000_‐SiNcTI‐Ph/CpG‐ZIF‐8@CM). H) TEM images of PEG_2000_‐SiNcTI‐Ph/CpG‐ZIF‐8 and PEG_2000_‐SiNcTI‐Ph/CpG‐ZIF‐8@CM. Scale bar, 100 nm. I,J) SDS‐PAGE protein analysis (I) and WB analysis (J) of PD‐1 and CD47 protein expression of HEK293T‐PD‐1 (1), HM (2), RBC (3), RM (4), and PEG_2000_‐SiNcTI‐Ph/CpG‐ZIF‐8@CM (5). K) The size variation of PEG_2000_‐SiNcTI‐Ph/CpG‐ZIF‐8@CM as a function of time for 8 days in PBS. Data are presented as mean ± S.D. (n = 3 independent experiments per group).

Phthalocyanine derivatives as highly efficient PS have been applied to many clinical PDT treatment of tumors.^[^ ^40^ ^]^ Although their maximum absorption red‐shifted by ≈100 nm compared with those of phthalocyanines, naphthalocyanine derivatives as PS showed limited application in PDT because of their poor solubility and strong aggregation.^[^ ^41^ ^]^ In our previous work, alkyl chains substituted naphthalocyanine tetraimides (SiNcTI‐N and SiNcTI‐Br) with excellent stability and slight aggregation were reported as cathode interlayer materials in organic solar cells, however they displayed little aqueous solubility, making their PDT usage impossible.^[^ ^42^ ^]^ To increase their aqueous solubility, we modified the compounds with different hydrophilic groups (i.e., SiNcTI‐NO, SiNcTI‐TFSI, SiNcTI‐2N, and SiNcTI‐2I) (Figure S1, Supporting Information), but frustratedly they still possessed limited aqueous solubility. Delightedly, when four isopropylphenyl groups and two methoxy polyethylene glycols chains (i.e., mPEG_1000_, mPEG_1500_, or mPEG_2000_) were introduced to naphthalocyanine tetraimides simultaneously, sufficient aqueous solubility was achieved. Compared with PEG_1000_‐SiNcTI‐Ph and PEG_1500_‐SiNcTI‐Ph, PEG_2000_‐SiNcTI‐Ph (**Figure** **1**A (left picture); Figures S2–S9, Supporting Information) demonstrated the best solubility and well dispersion in aqueous solution, which was chosen for following studies. The maximum absorption of PEG_2000_‐SiNcTI‐Ph was located at 790 nm (Figure 1A (right picture)), much longer than that of conventional commercial PSs, such as Ce6 (650 nm) and rose Bengal (530 nm). The high‐efficiency long‐wavelength photon sensitization of PEG_2000_‐SiNcTI‐Ph was confirmed by the fluorescence increase of 2,7‐dichlorodihydronflurescein diacetate (DCFH‐DA) solution and adsorption decrease of 1,3‐diphenylisobenzofuran (DPBF) solution under 808 nm laser irradiation, indicating the generation of ROS (Figure 1B,C; Figures S10 and S11 Supporting Information).

Next, PEG_2000_‐SiNcTI‐Ph and CpG were co‐embedded within ZIF‐8 in one step during the self‐assembly of the metal‐organic framework, with the loading efficiency up to 46.6% for PEG_2000_‐SiNcTI‐Ph. The obtained PEG_2000_‐SiNcTI‐Ph/CpG‐ZIF‐8 nanocomposite could be well dispersed in PBS, guaranteeing the premise for in vitro and in vivo applications (Figure 1D). In addition, after encapsulation, the content of CpG in centrifugal supernatant decreased (Figure S12, Supporting Information) and agarose gel electrophoresis directly confirmed the success of CpG loading (Figure S13, Supporting Information). The observation by scanning electron microscopy (SEM) illustrated that PEG_2000_‐SiNcTI‐Ph/CpG‐ZIF‐8 demonstrated the spherical structure with an average size of ≈150 nm (Figure S14, Supporting Information). Meanwhile, X‐ray diffraction (XRD) measurement revealed no significant difference in the crystal structure and crystallinity among ZIF‐8, PEG_2000_‐SiNcTI‐Ph‐ZIF‐8, and PEG_2000_‐SiNcTI‐Ph/CpG‐ZIF‐8, indicating that the embedding of PEG_2000_‐SiNcTI‐Ph and CpG barely affected the formation of ZIF‐8 (Figure S15, Supporting Information).

Eventually, PEG_2000_‐SiNcTI‐Ph/CpG‐ZIF‐8 was encapsulated by the CM consisting of HM and RM. To obtain a reliable alternative for immune checkpoint PD‐L1 blockade to advance anti‐tumor immune response, we transfected PD‐1 plasmifd (C‐GFPSpark tag) into HEK293T cells. Fluorescence imaging (Figure 1E) and western blot (WB) analyses (Figure S16, Supporting Information) confirmed the successful transfection of the plasmid and expression of PD‐1 on HEK293T cells. Likewise, flow cytometry detection verified the existence of typical markers PD‐1 (35%) and CD47 (18%) proteins expressed on HEK293T‐PD‐1 cells and RBC, respectively (Figure 1F). HM and RM were then extracted from HEK293T‐PD‐1 cells and RBC, respectively, and co‐extruded with PEG_2000_‐SiNcTI‐Ph/CpG‐ZIF‐8 to obtain PEG_2000_‐SiNcTI‐Ph/CpG‐ZIF‐8@CM. Dynamic light scattering (DLS) results showed a slight increase in the particle size of PEG_2000_‐SiNcTI‐Ph/CpG‐ZIF‐8 after CM encapsulation (Figure S17, Supporting Information). The shift of the zeta potential of the nanocomposites from positive to negative charge also indicated the successful encapsulation by CM (Figure 1G). Worth mentioning, the decrease of zeta potential of PEG_2000_‐SiNcTI‐Ph‐ZIF‐8 after embedding with CpG was because of the anionic attribute of CpG. And the corresponding morphological observation by transmission electron micrographs (TEM) directly revealed the thickness of the outer membrane of PEG_2000_‐SiNcTI‐Ph/CpG‐ZIF‐8@CM was ≈10 nm (Figure 1H). Furthermore, as can be noted from the sodium dodecyl sulfate‐polyacrylamide gel electrophoresis (SDS‐PAGE) results, HM retained most of the proteins of HEK293T‐PD‐1 cells and RM retained major proteins of RBCs. Similarly, it can be observed that some proteins which might exist in the cytoplasm disappeared from the cell membrane compared to the cells. As expected, major proteins on HM and RM were successfully reserved on PEG_2000_‐SiNcTI‐Ph/CpG‐ZIF‐8@CM (Figure 1I). Among these proteins, the functional proteins PD‐1 and CD47 expressed on HEK293T‐PD‐1 and RBC, respectively, were crucial for achieving immune checkpoint PD‐L1 blockade/tumor targeting^[^ ^33^ ^]^ and evasion of macrophage phagocytosis.^[^ ^32^ ^]^ WB results exhibited that PEG_2000_‐SiNcTI‐Ph/CpG‐ZIF‐8@CM well preserved PD‐1 and CD47 proteins from parental cells, laying down the foundation for empowering the photodynamic immunotherapy (Figure 1J). In addition, there was no obvious variation in the particle size of PEG_2000_‐SiNcTI‐Ph/CpG‐ZIF‐8@CM in PBS over the course of 8 days, indicating the superior stability of PEG_2000_‐SiNcTI‐Ph/CpG‐ZIF‐8@CM under physiological environment (Figure 1K).

### 2.2 Tumor Environment‐Activated Photodynamic Effect of PEG_2000_‐SiNcTI‐Ph/CpG‐ZIF‐8@CM

**Figure 2:**
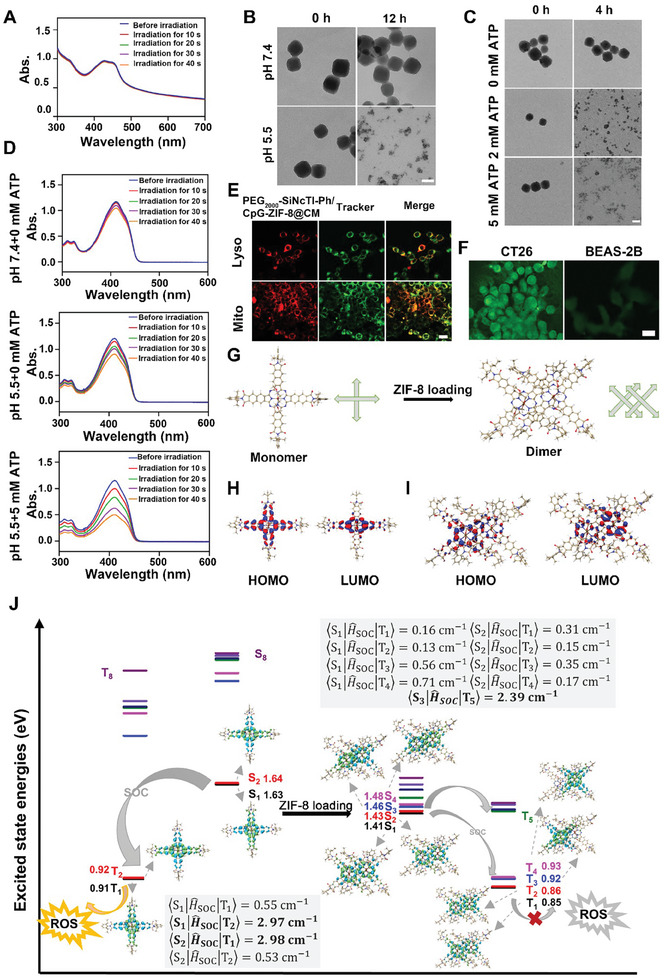
PEG_2000_‐SiNcTI‐Ph/CpG‐ZIF‐8@CM demonstrated tumor environment‐activated photodynamic effect and its underlying mechanism. A) Evaluation of ROS generation ability of PEG_2000_‐SiNcTI‐Ph/CpG‐ZIF‐8@CM under 808 nm laser irradiation measured by DPBF. B) TEM images of PEG_2000_‐SiNcTI‐Ph/CpG‐ZIF‐8@CM in PBS (pH 7.4 or 5.5) for 0 and 12 h, respectively. Scale bar, 100 nm. C) TEM images of PEG_2000_‐SiNcTI‐Ph/CpG‐ZIF‐8@CM incubated with different concentrations of ATP for 4 h. Scale bar, 100 nm. D) ROS generation ability of PEG_2000_‐SiNcTI‐Ph/CpG‐ZIF‐8@CM incubated in PBS with different pH and/or with different concentrations of ATP for 4 h measured by DPBF. E) CLSM images of PEG_2000_‐SiNcTI‐Ph/CpG‐ZIF‐8@CM localization in lysosomes and mitochondria. Scale bar, 20 µm. Red: PEG_2000_‐SiNcTI‐Ph/CpG‐ZIF‐8@CM were labeled by Cy5. Green: Lyso Tracker Green DND‐26 or Mito Tracker Green carbocyanine. F) CLSM images of CT26 cells/ BEAS‐2B cells stained with DCFH‐DA after incubation with PEG_2000_‐SiNcTI‐Ph/CpG‐ZIF‐8@CM under 808 nm laser irradiation. Scale bar, 20 µm. G) Optimized ground‐state (S_0_) geometries of monomer (left) and dimer (right) at the B3LYP‐GD3BJ/6‐31G (d) level. H,I) The HOMO and LUMO distribution plots for monomer (H) and dimer (I). J) Theoretical explanations for the ROS generation by PEG_2000_‐SiNcTI‐Ph and dormancy of ROS generation capacity of PEG_2000_‐SiNcTI‐Ph/CpG‐ZIF‐8@CM.

Unexpectedly, we found that the photodynamic feature of PEG_2000_‐SiNcTI‐Ph vanished after being embedded into ZIF‐8 framework (**Figure** **2**A). This phenomenon enlightened us if dormancy of the photodynamic effect of PEG_2000_‐SiNcTI‐Ph could be reversed once the release of PEG_2000_‐SiNcTI‐Ph was triggered after the degradation of ZIF‐8 framework within the tumor due to its sensitivity to tumor‐specific stresses of low pH and high concentrations of ATP,^[^ ^43^, ^44^, ^45^ ^]^ a tumor environment‐activated photodynamic effect would be achieved to realize precise cancer therapy. And following evaluations were performed to verify the assumption. First, the morphology of PEG_2000_‐SiNcTI‐Ph/CpG‐ZIF‐8@CM after incubation in PBS at different pH or with different concentrations of ATP showed that PEG_2000_‐SiNcTI‐Ph/CpG‐ZIF‐8@CM was selectively degraded in acidic or ATP‐enriched conditions (Figure 2B,C), which contributed to the gradual release of PEG_2000_‐SiNcTI‐Ph, eventually reaching as high as >80% after 24 h, in a striking contrast to the negligible release within conditions of pH = 7.4 or 0 mM ATP (Figure S18, Supporting Information). Meantime, when PEG_2000_‐SiNcTI‐Ph/CpG‐ZIF‐8@CM was incubated in PBS with low pH (5.5) plus high concentrations of ATP, its particle size decreased over time, further indicating that PEG_2000_‐SiNcTI‐Ph/CpG‐ZIF‐8@CM was degraded gradually (Figure S19, Supporting Information). Excitingly, a recovery of ROS generation ability was noticed after the release of PEG_2000_‐SiNcTI‐Ph under 808 nm laser irradiation. As expected, compared with the action of low pH or high concentrations of ATP alone, under their simultaneous stimuli mimicking tumor environment, ZIF‐8 degraded the fastest to release the most PEG_2000_‐SiNcTI‐Ph, thus demonstrating the most significant recovery of ROS generation (Figure 2D; Figures S20–S24, Supporting Information), which was in stark contrast to the feeble photodynamic effect of PEG_2000_‐SiNcTI‐Ph/CpG‐ZIF‐8@CM within conditions of pH = 7.4 plus 0 mM ATP approaching normal physiological environment, laying down the foundation for tumor environment‐activated PDT beneficial for the precise treatment of colon cancer.

Next, the ROS generation ability of PEG_2000_‐SiNcTI‐Ph/CpG‐ZIF‐8@CM at the cellular level was evaluated to determine their tumor environment‐activated photodynamic efficacy in vitro. The intracellular uptake and distribution showed that the red fluorescence from Cy5‐labeled PEG_2000_‐SiNcTI‐Ph/CpG‐ZIF‐8@CM was highly co‐localized with the green fluorescence from Lyso Tracker or Mito Tracker, indicating that the nanocomposites could be effectively engulfed by CT26 tumor cells and accumulated within lysosome and mitochondria, where acids and ATP were enriched, respectively (Figure 2E).^[^ ^25^, ^26^ ^]^ With above evidence supported, then, we evaluated the ROS generation using DCFH‐DA probe at the cellular level by confocal laser scanning microscopy (CLSM). As shown in Figure 2F, CT26 tumor cells incubated with PEG_2000_‐SiNcTI‐Ph/CpG‐ZIF‐8@CM exhibited much brighter green fluorescence under 808 nm laser irradiation compared with those incubated with normal human lung epithelial cells BEAS‐2B, which should be attributed to that the higher concentrations of ATP^[^ ^43^ ^]^ plus lower pH^[^ ^44^, ^45^ ^]^ within CT26 tumor cells than BEAS‐2B normal cells promoted the release of PEG_2000_‐SiNcTI‐Ph to recover the photodynamic effect.

In terms of above observed phenomenon, we suspected that PEG_2000_‐SiNcTI‐Ph would form an aggregated state after being loaded within ZIF‐8, which hindered the ability of PEG_2000_‐SiNcTI‐Ph to generate ROS. Then, we deciphered the underlying mechanism by which PEG_2000_‐SiNcTI‐Ph/CpG‐ZIF‐8@CM demonstrated tumor environment‐activated photodynamic effect through quantum chemical calculations. First, we compared the electron paramagnetic resonance (EPR) signals of PEG_2000_‐SiNcTI‐Ph under liquid (dispersed state) and solid (aggregated state) conditions (Figure S25, Supporting Information). The results showed the PEG_2000_‐SiNcTI‐Ph molecules were more effective in generating ROS when they were in the dispersed state. To simplify the computational model, we used the monomer and dimer models to represent the unassembled and aggregated states of PEG_2000_‐SiNcTI‐Ph, respectively (Figure 2G). Overall, based on the distribution of highest occupied molecular orbitals (HOMOs) and lowest unoccupied molecular orbitals (LUMOs), both the singlet and triplet states showed a typical π → π* local excitation character along the horizontal axis of molecular skeleton partly mixed with a π → π* charge transfer character along their vertical axes (Figure 2H,I). Interestingly, the time‐dependent density functional theory (TDDFT) calculations suggested that the two lowest singlet excited states (S_1_ and S_2_) of the PEG_2000_‐SiNcTI‐Ph molecule possessed almost the same (degenerate) energy levels, indicating the natural advantage of such a molecular configuration having twice as many channels of photosensitization. Due to the strong spin‐orbit coupling (SOC) interactions between the singlet (S) and triplet (T) excited states, the intersystem crossing (ISC) process between S and T became stronger and they mainly occurred through S1/T2 and S2/T1 channels with significantly large SOC values of ≈3 cm^−1^, which benefits enhancing the reactivity and opening the ROS channel (Figure 2J; Figure S26, Supporting Information). In the dimer model, the ISC process between S and T was extremely attenuated. Although a large SOC value for the S3/T5 channel existed, the large energy gap between T5 and T1‐T4 prevented the possible internal conversion process. Therefore, the aggregated molecules of the triplet state were unable to generate ROS sensitized by long‐wavelength photons, or the ability to generate ROS was greatly weakened (Figure 2J; Figure S27, Supporting Information). Collectively, these findings suggested the rational design of the molecular structure of PSs and ingenious modulation of their delivery state are essential for the realization of tumor environment‐activated photodynamic effect.

### 2.3 PEG_2000_‐SiNcTI‐Ph/CpG‐ZIF‐8@CM‐Based Tumor Environment‐Activated PDT Induced Efficient Tumor‐Associated Immune Response

**Figure 3:**
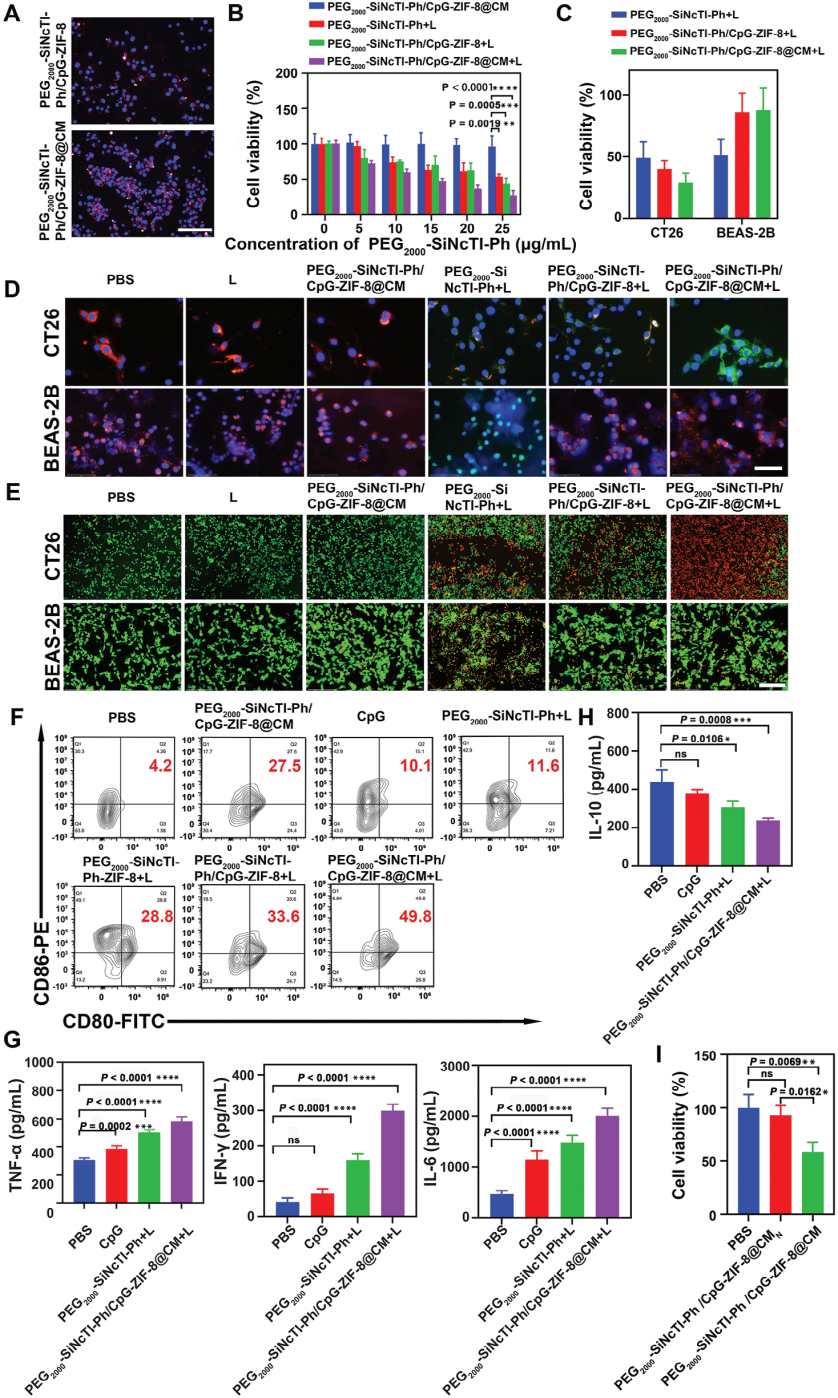
PEG_2000_‐SiNcTI‐Ph/CpG‐ZIF‐8@CM‐based tumor environment‐activated PDT advanced anti‐tumor immune response in vitro. A) Fluorescence images of CT26 cells after incubation with PEG_2000_‐SiNcTI‐Ph/CpG‐ZIF‐8 or PEG_2000_‐SiNcTI‐Ph/CpG‐ZIF‐8@CM. Scale bar, 150 µm. B) CT26 cell viabilities after different concentrations of different nanocomposites treatments with (+L) or without 808 nm laser irradiation. C) CT26 and BEAS‐2B cell viabilities after different nanocomposites treatments with (+L) 808 nm laser irradiation. D) Fluorescence images of CT26 and BEAS‐2B cells stained with JC‐1 under different treatments to determine the variation of MMP. Scale bar, 75 µm. E) Live/dead staining of CT26 and BEAS‐2B cells after different treatments. Scale bar, 275 µm. F) Flow cytometric analysis of the expression levels of mature DC markers CD80/CD86 (Q2 quadrant represents the percentage ratio of mature DC) in splenic immune cells co‐cultured with supernatants of CT26 cells after different treatments. G,H) Secretion levels of immunoregulatory cytokines, including TNF‐α, IFN‐γ, IL‐6 (G), and IL‐10 (H) in splenic immune cells co‐incubated with supernatants of CT26 cells after different treatments. I) Rescue of PD‐L1‐mediated T cell exhaustion by PEG_2000_‐SiNcTI‐Ph/CpG‐ZIF‐8@CM in co‐culturing tumor cells and IL‐2‐activated T cells. Statistical analysis was calculated via one‐way ANOVA. ^*^ *p* <0.05, ^**^ *p* <0.01, ^***^ *p *<0.001, and ^****^ *p* <0.0001. “ns” represents no significant difference. Data are presented as mean ± S.D. (n = 3 biologically independent experiments per group).

With aforementioned superiority of tumor environment‐activated photodynamic effect of PEG_2000_‐SiNcTI‐Ph/CpG‐ZIF‐8@CM, we began to evaluate PEG_2000_‐SiNcTI‐Ph/CpG‐ZIF‐8@CM‐based tumor environment‐activated PDT in vitro. First, the negligible cytotoxicity of PEG_2000_‐SiNcTI‐Ph/CpG‐ZIF‐8@CM was identified (Figure S28, Supporting Information). Subsequently, the cell uptake assay revealed a stronger red fluorescence in the cytoplasm of CT26 cells treated with Cy5‐labeled PEG_2000_‐SiNcTI‐Ph/CpG‐ZIF‐8@CM compared with that treated with Cy5‐labeled PEG_2000_‐SiNcTI‐Ph/CpG‐ZIF‐8 owing to the PD‐1/PD‐L1‐mediated endocytosis, beneficial for tumor environment‐activated PDT (**Figure** **3**A). After the nanocomposite entered the tumor cell and responded the abundant acids plus rich ATP to release PEG_2000_‐SiNcTI‐Ph, the recovery of strong ROS generation capacity would possess an outstanding potential to kill tumor cells. Under 808 nm laser irradiation, PEG_2000_‐SiNcTI‐Ph/CpG‐ZIF‐8@CM‐based tumor environment‐activated PDT could significantly induce more CT26 tumor cell death than the PEG_2000_‐SiNcTI‐Ph‐ or PEG_2000_‐SiNcTI‐Ph/CpG‐ZIF‐8‐based PDT under the same applied concentration of PEG_2000_‐SiNcTI‐Ph (Figure 3B). Above‐verified different ROS generation ability of PEG_2000_‐SiNcTI‐Ph/CpG‐ZIF‐8@CM under laser irradiation within tumor and normal cells encouraged us to evaluate the selective killing effect of PEG_2000_‐SiNcTI‐Ph/CpG‐ZIF‐8@CM toward tumor cells under laser irrdiation, of which the attribute would lower unnecessary damages toward normal cells to the greatest extent, advancing the precision of cancer therapy. The results of CT26 tumor and BEAS‐2B normal cell viabilities evaluated by CCK‐8 assay showed that under laser irradiation, PEG_2000_‐SiNcTI‐Ph inhibited the two cell proliferation by ≈50%, while PEG_2000_‐SiNcTI‐Ph/CpG‐ZIF‐8 and PEG_2000_‐SiNcTI‐Ph/CpG‐ZIF‐8@CM exhibited the negligible inhibition on BEAS‐2B normal cells, in a striking contrast to the inhibition rates of 60% and 71% against CT26 tumor cells, respectively (Figure 3C). Besides, the generated ROS could also cause changes in mitochondrial membrane potential (MMP) to induce cell death.^[^ ^46^ ^]^ The variation from red fluorescence of JC‐1 aggregates to green fluorescence of JC‐1 monomers indicated that PEG_2000_‐SiNcTI‐Ph/CpG‐ZIF‐8@CM‐based tumor environment‐activated PDT led to the most obvious MMP variation, which would promote CT26 tumor cell death. For BEAS‐2B normal cells, PEG_2000_‐SiNcTI‐Ph caused cellular mitochondrial damage under laser irradiation evidenced by the appearance of green fluorescence. However, the damage was avoided by loading PEG_2000_‐SiNcTI‐Ph into ZIF‐8 as indicated by no green fluorecence appearing within BEAS‐2B normal cells treated with PEG_2000_‐SiNcTI‐Ph/CpG‐ZIF‐8 or PEG_2000_‐SiNcTI‐Ph/CpG‐ZIF‐8@CM under laser irradiaiton (Figure 3D). In addition, as expected, the live/dead staining assay visually revealed that a significant amount of red fluorescence from propidium iodide (PI)‐stained dead cells was observed for CT26 tumor cells treated with PEG_2000_‐SiNcTI‐Ph/CpG‐ZIF‐8@CM under laser irradiation. Whereas, there was only a small amount of cell death happening for BEAS‐2B normal cells treated with PEG_2000_‐SiNcTI‐Ph/CpG‐ZIF‐8@CM under laser irradiation, which suggested that PEG_2000_‐SiNcTI‐Ph/CpG‐ZIF‐8@CM selectively caused damage to tumor cells and demonstrated negligible toxicity to normal cells under laser irradiation, consistent with above CCK‐8 and MMP evalulation assays, validating the tumor environment‐activated PDT realized by PEG_2000_‐SiNcTI‐Ph/CpG‐ZIF‐8@CM (Figure 3E).

PDT could induce ICD^[^ ^47^ ^]^ to release damage‐associated molecular patterns (DAMPs), such as calreticulin (CRT), high mobility group protein 1 (HMGB1), and heat shock protein (HSP70), to maturate DC for the activation of immune response, which would advance the anti‐tumor effect. The WB analysis demonstrated that under laser irradiation for CT26 tumor cells, PEG_2000_‐SiNcTI‐Ph/CpG‐ZIF‐8@CM induced more expression/release of CRT, HMGB1, and HSP70 (Figure S29, Supporting Information). Subsequently, a co‐culturing system of splenic immune cells and supernatants of CT26 tumor cells after different treatments was constructed to evaluate how the immune system reacted to ICD. The flow cytometry analysis showed that the percentages of CD80/CD86 double‐positive DC was the highest for PEG_2000_‐SiNcTI‐Ph/CpG‐ZIF‐8@CM‐based tumor environment‐activated PDT, reaching as high as ≈49.8%. It was worth mentioning that the percentages of CD80/CD86 double‐positive DC in the PEG_2000_‐SiNcTI‐Ph/CpG‐ZIF‐8@CM group was 27.5%, whereas CpG alone cannot elicit considerable non‐specific immunity (only ≈10.1%) due to the electro‐repulsive force between negatively charged cell membrane and anionic CpG, illustrating the rationality of our design to load CpG to enhance its DC uptake to promote the maturation of DCs for boosting the tumor‐associated immune response (Figure 3F). Furthermore, these mature DCs would then present tumor‐associated antigens to native T cells, which would differentiate into cytotoxic CD8^+^ T cells to elicit immune response for the anti‐tumor purpose.^[^ ^48^ ^]^ As a result, PEG_2000_‐SiNcTI‐Ph/CpG‐ZIF‐8@CM‐based tumor environment‐activated PDT led to the highest activation of CD8^+^ T cells (Figure S30, Supporting Information). Correspondingly, we investigated the cytokine secretion levels by enzyme‐linked immunosorbent assay (ELISA). The results showed that the levels of pro‐inflammatory cytokines (Figure 3G), such as tumor necrosis factor‐α (TNF‐α), interferon‐γ (IFN‐γ), and interleukin‐6 (IL‐6), were significantly increased, while the secreted level of the anti‐inflammatory cytokine (Figure 3H) interleukin‐10 (IL‐10) was significantly reduced when PEG_2000_‐SiNcTI‐Ph/CpG‐ZIF‐8@CM‐based tumor environment‐activated PDT was applied rather than PEG_2000_‐SiNcTI‐Ph‐based treatment or CpG treatment alone. These results suggested that PEG_2000_‐SiNcTI‐Ph/CpG‐ZIF‐8@CM‐based tumor environment‐activated PDT could effectively activate DCs and induce robust immune response by combining non‐specific and specific immunities.

However, in most case, immune response for the anti‐tumor effect would be severely attenuated due to the self‐protection mechanism of tumor cells against activated T cells, e.g., PD‐1/PD‐L1 interaction. Then, we further evaluated the ability of PEG_2000_‐SiNcTI‐Ph/CpG‐ZIF‐8@CM for immune checkpoint PD‐L1 blockade to empower anti‐tumor immune response. For direct comparison, PEG_2000_‐SiNcTI‐Ph/CpG‐ZIF‐8@CM_N_, which was encapsulated by the fusion cell membrane consisting of un‐transfected HEK293T cell membranes and RM, was fabricated and applied along with PEG_2000_‐SiNcTI‐Ph/CpG‐ZIF‐8@CM for incubation with CT26 cells, respectively, followed by the addition of the interleukin‐2 (IL‐2)‐activated T cells (cytotoxic T cells). As shown in Figure 3I, compared with the PEG_2000_‐SiNcTI‐Ph/CpG‐ZIF‐8@CM_N_, the CT26 cell viability with PEG_2000_‐SiNcTI‐Ph/CpG‐ZIF‐8@CM incubation decreased significantly (*P* = 0.0162), indicating that immune checkpoint PD‐L1 blockade by PEG_2000_‐SiNcTI‐Ph/CpG‐ZIF‐8@CM restored the cytotoxic activity of T cells to advance the anti‐tumor effect.

### 2.4 In Vivo Therapeutic Efficacy of PEG_2000_‐SiNcTI‐Ph/CpG‐ZIF‐8@CM‐Based Tumor Environment‐Activated PDT

**Figure 4:**
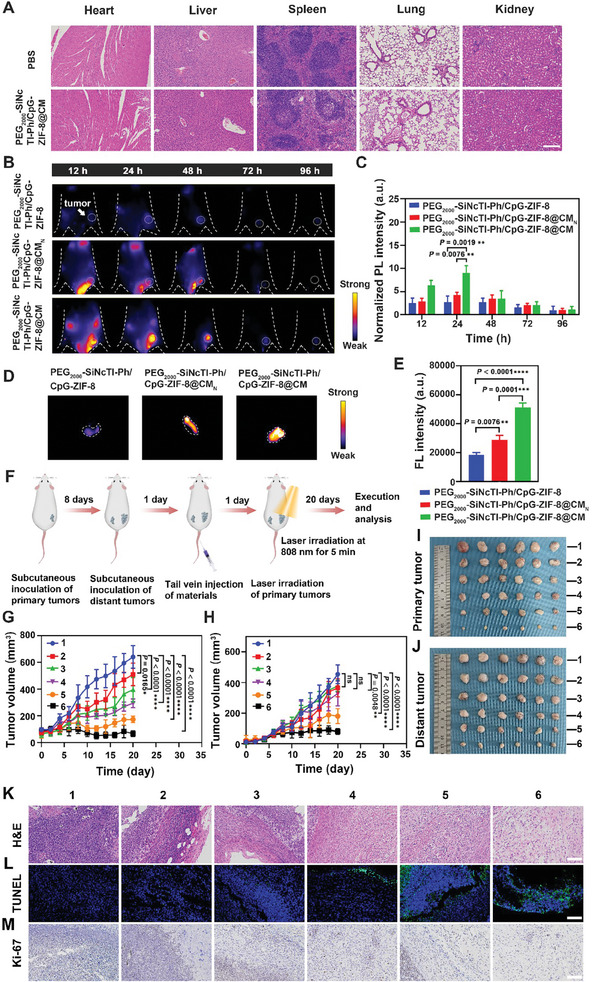
In vivo pharmacokinetics and therapeutic effect of PEG_2000_‐SiNcTI‐Ph/CpG‐ZIF‐8@CM‐based tumor environment‐activated PDT in CT26 tumor‐bearing mice. A) H&E staining images of heart, liver, spleen, lung, and kidney from the mice with or without PEG_2000_‐SiNcTI‐Ph/CpG‐ZIF‐8@CM administration. Scale bar, 200 µm. B) Time‐dependent fluorescence imaging of CT26 tumor‐bearing mice intravenously injected with different materials. C) The quantitative analysis of fluorescence intensity based on (B). D) Ex vivo fluorescence images of tumor tissues of mice at 24 h post intravenous administration of different materials. E) The quantitative analysis of fluorescence intensity of ex vivo tumor tissues of mice at 24 h post intravenous administration based on (D). F) Schematic illustration of the therapeutic procedure. G,H) The volume variation of primary (G) and distant tumors (H) as a function of time among different treatments. I,J) Photograph of harvested primary (I) and distant (J) tumors from mice of different treatment groups after 20 days' treatment. K‐M) H&E staining (K), TUNEL staining (L), and Ki‐67 immunohistochemical staining (M) of collected tumors from different treatment groups. Scale bar, 100 µm; (Green: TUNEL was labeled by FITC; Blue: Cell nuclei were stained by DAPI). Statistical analysis was calculated via one‐way ANOVA.^*^ *p* <0.05, ^**^ *p* <0.01, ^***^ *p* <0.001, and ^****^ *p *<0.0001. “ns” represents no significant difference. Data are presented as mean ± S.D. (n = 3 (C, E) or 6 (G, H) biologically independent experiments per group). (1: PBS, 2: PEG_2000_‐SiNcTI‐Ph/CpG‐ZIF‐8@CM, 3: PEG_2000_‐SiNcTI‐Ph+L, 4: PEG_2000_‐SiNcTI‐Ph‐ZIF‐8+L, 5: PEG_2000_‐SiNcTI‐Ph/CpG‐ZIF‐8+L, 6: PEG_2000_‐SiNcTI‐Ph/CpG‐ZIF‐8@CM+L; “L” represents the laser irradiation.).

Above satisfactory in vitro evaluations motiviated us to further perform in vivo studies. The in vivo biosafety of the nanocomposite was first evaluated, of which the superior biocompatibility of PEG_2000_‐SiNcTI‐Ph/CpG‐ZIF‐8@CM was revealed by no significant differences of blood histopathological analysis (i.e., indicators of liver and kidney injuries, lipids, blood glucose, and cardiac enzyme profiles) and hematoxylin‐eosin (H&E) staining of tissues among mice with and without administration of PEG_2000_‐SiNcTI‐Ph/CpG‐ZIF‐8@CM (**Figure** **4**A; Figures S31–S34, Supporting Information).

The CD47 expressed on PEG_2000_‐SiNcTI‐Ph/CpG‐ZIF‐8@CM could facilitate the delivery efficacy by evasion of macrophage surveillance. To evaluate this, Cy5‐labeled PEG_2000_‐SiNcTI‐Ph/CpG‐ZIF‐8 and Cy5‐labeled PEG_2000_‐SiNcTI‐Ph/CpG‐ZIF‐8@CM were incubated with RAW264.7 macrophages for 2 h, respectively. The results showed that compared with the former, the latter exhibited weaker red fluorescence, indicating that the PEG_2000_‐SiNcTI‐Ph/CpG‐ZIF‐8@CM was less phagocytosed by RAW264.7 cells (Figure S35, Supporting Information). To further explore this potential in vivo, mouse blood specimens after administration of different kinds of nanomaterials (i.e., PEG_2000_‐SiNcTI‐Ph, PEG_2000_‐SiNcTI‐Ph/CpG‐ZIF‐8, and PEG_2000_‐SiNcTI‐Ph/CpG‐ZIF‐8@CM) were collected for fluorescence imaging and spectral analysis based on the autofluorescence of PEG_2000_‐SiNcTI‐Ph (Figure S36, Supporting Information). Compared with the blood half‐life of 12 min and 19 min for PEG_2000_‐SiNcTI‐Ph and PEG_2000_‐SiNcTI‐Ph/CpG‐ZIF‐8, respectively, PEG_2000_‐SiNcTI‐Ph/CpG‐ZIF‐8@CM exhibited the longest blood circulation with blood half‐life time of 33 min (Figure S37, Supporting Information). Similarly, compared to PEG_2000_‐SiNcTI‐Ph and PEG_2000_‐SiNcTI‐Ph/CpG‐ZIF‐8 groups, the mean retention time (MRT) and area under curve (AUC) were longer and larger than those in the PEG_2000_‐SiNcTI‐Ph/CpG‐ZIF‐8@CM group, respectively, indicating the improved pharmacokinetics of PEG_2000_‐SiNcTI‐Ph/CpG‐ZIF‐8@CM (Figure S38, Supporting Information). The prolonged blood circulation and PD‐L1/PD‐1 interaction‐mediated endocytosis as verified above should lead to more accumulation of PEG_2000_‐SiNcTI‐Ph/CpG‐ZIF‐8@CM within the tumor over time to favor the following therapeutic effect. As expected, compared with PEG_2000_‐SiNcTI‐Ph/CpG‐ZIF‐8 and PEG_2000_‐SiNcTI‐Ph/CpG‐ZIF‐8@CM_N_, the fluorescence intensity at the tumor site was the strongest for PEG_2000_‐SiNcTI‐Ph/CpG‐ZIF‐8@CM‐adminstrated CT26 tumor‐bearing mice (Figure 4B,C), saturating at 24 h post administration, which also got supported by the ex vivo imaging results (Figure 4D,E).

The in vivo tumor targeting effect of PEG_2000_‐SiNcTI‐Ph/CpG‐ZIF‐8@CM will advance the precise anti‐tumor effect. Next, we systematically evaluated the anti‐tumor ability of PEG_2000_‐SiNcTI‐Ph/CpG‐ZIF‐8@CM‐based tumor environment‐activated PDT. As illustrated in Figure 4F, the bilateral CT26 tumor model was first established to simulate primary and distant tumors. Guided by the fluorescence imaging‐based biodistribution results, 808 nm laser irradiation was performed for the primary tumor at 24 h post‐injection of different materials (denoted as 1: the PBS group, 2: the PEG_2000_‐SiNcTI‐Ph/CpG‐ZIF‐8@CM group, 3: the PEG_2000_‐SiNcTI‐Ph+L group, 4: the PEG_2000_‐SiNcTI‐Ph‐ZIF‐8+L group, 5: the PEG_2000_‐SiNcTI‐Ph/CpG‐ZIF‐8+L group, 6: the PEG_2000_‐SiNcTI‐Ph/CpG‐ZIF‐8@CM+L group; “L” represented laser irradiation). Due to the aggressive nature of CT26 tumors, the primary and distant tumor growth in the PBS group was rapid. Intriguingly, the tumor growth in the PEG_2000_‐ SiNcTI‐Ph/CpG‐ZIF‐8+L group was more inhibited than that in the PEG_2000_‐SiNcTI‐Ph‐ZIF‐8+L group, which was attributed to the CpG‐induced non‐specific immunity. Likewise, without laser irradiation, PEG_2000_‐SiNcTI‐Ph/CpG‐ZIF‐8@CM also exhibited slight tumor suppression induced by non‐specific immunity resulting from the nanoplatform delivery of CpG. Importantly, a more obvious tumor‐inhibiting effect was observed from the PEG_2000_‐SiNcTI‐Ph/CpG‐ZIF‐8@CM+L group (Figure 4G; Figure S39, Supporting Information). In addition, excised primary tumors in the PEG_2000_‐SiNcTI‐Ph/CpG‐ZIF‐8@CM+L group were the smallest after 20 days of treatment, down to 67.34 ± 21.86 mm^3^ (*p* <0.0001) with tumor size from the PBS, PEG_2000_‐SiNcTI‐Ph/CpG‐ZIF‐8@CM, PEG_2000_‐SiNcTI‐Ph+L, PEG_2000_‐SiNcTI‐Ph‐ZIF‐8+L, and PEG_2000_‐SiNcTI‐Ph/CpG‐ZIF‐8+L groups being 641.94 ± 84.93 mm^3^, 511.22 ± 84.61 mm^3^ (*P* = 0.0165), 397.32 ± 92.25 mm^3^ (*p* <0.0001), 294.67 ± 32.35 mm^3^ (*p* <0.0001), and 174.44 ± 24.27 mm^3^ (*p* <0.0001), respectively (Figure 4I). It was worth noting that the volume of distant tumors in the PEG_2000_‐SiNcTI‐Ph/CpG‐ZIF‐8@CM+L group was also significantly suppressed, which should be contributed by the combination of anti‐tumor immune response elicited by tumor environment‐activated PDT (this also got validated in the following part), immune checkpoint PD‐L1 blockade, and CpG‐enhanced immunity as characterized above (Figure 4H,J; Figure S40, Supporting Information). Moreover, during the whole treatment period, no significant body weight loss was observed in all groups, indicating that the nanoplatforms demonstrated negligible side effects (Figure S41, Supporting Information).

To further confirm the therapeutic effect, H&E staining (Figure 4K), Terminal deoxynucleotidyl transferase dUTP nick end labeling (TUNEL) staining (Figure 4L), and Ki‐67 immunohistochemical staining (Figure 4M) were used for histological examination of tumors. H&E staining showed the severest cell death with irregular cell shapes and nuclei atrophy in the PEG_2000_‐SiNcTI‐Ph/CpG‐ZIF‐8@CM+L group compared with other groups. Supported by TUNEL staining and Ki‐67 immunohistochemical staining, the severest cell death of tumor cells and the least cell proliferation were observed in the PEG_2000_‐SiNcTI‐Ph/CpG‐ZIF‐8@CM+L group. All these results indicated that tumor environment‐activated PDT, the tumor non‐specific immunity induced by CpG, immune checkpoint PD‐L1 blockade, and prolonged blood circulation plus tumor targeting contributed by the CM coating together led to the satisfactory anti‐tumor effect.

### 2.5 PEG_2000_‐SiNcTI‐Ph/CpG‐ZIF‐8@CM‐Based Tumor Environment‐Activated PDT Reshaped the Immune Environment to Advance the Anti‐Tumor Effect and Inhibit Tumor Metastasis and Recurrence

**Figure 5:**
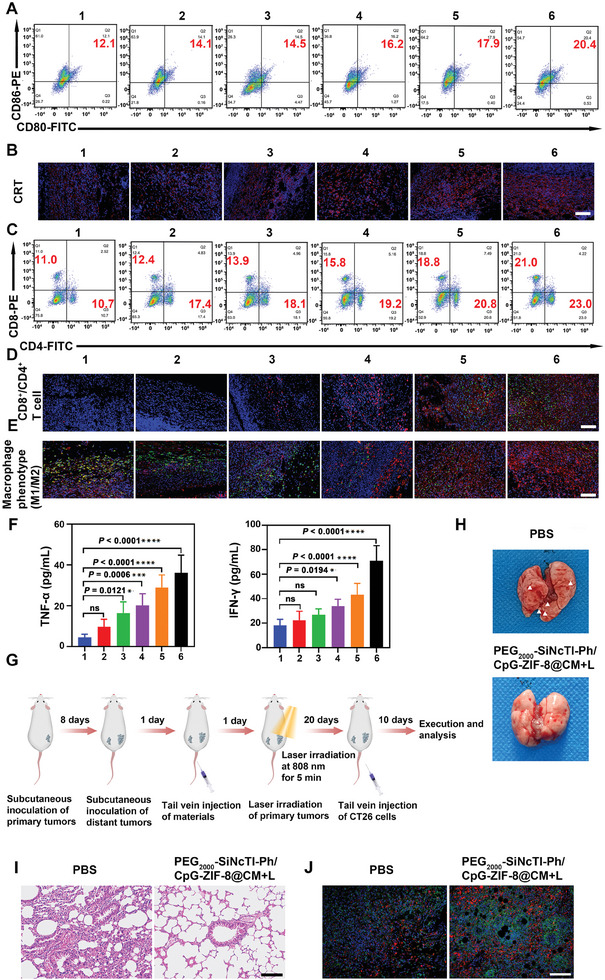
PEG_2000_‐SiNcTI‐Ph/CpG‐ZIF‐8@CM‐based tumor environment‐activated PDT induced effective immunotherapy of CT26 tumors. A) Flow cytometric analysis on the mature DC markers CD80/CD86 (Q2 quadrant represents the percentage ratio of mature DC) after different treatments. B) Immunofluorescence analysis on the expression of CRT on the tumors from CT26 tumor‐bearing mice after different treatments. Scale bar, 100 nm. Red: CRT was labeled by Cy3. Blue: Cell nuclei were stained by DAPI. C) Flow cytometric analysis on the T cell surface protein makers CD4^+^/CD8^+^ (Q1 and Q3 quadrants represent the percentage of CD4^+^ and CD8^+^ cells, respectively) after different treatments. D) Immunofluorescence analysis of CD4^+^/CD8^+^ T cell infiltration within primary tumors in CT26 tumor‐bearing mice after different treatments. Scale bar, 100 µm. Red: CD4 was labeled by Cy3. Green: CD8 was labeled by FITC. Blue: Cell nuclei were stained by DAPI. E) Immunofluorescence analysis of the M1‐ (iNOS positive) and M2‐ (CD206 positive) type macrophages within tumors after different treatments. Scale bar, 100 nm. Red: iNOS was labeled by Cy3. Green: CD206 was labeled by FITC. Blue: Cell nuclei were stained by DAPI. F) Peripheral blood cytokine levels of TNF‐α (left picture) and IFN‐γ (right picture) after different treatments. G) Schematic illustration for PEG_2000_‐SiNcTI‐Ph/CpG‐ZIF‐8@CM‐based treatment‐mediated inhibition of tumor metastasis and recurrence. H, I) Representative photographs (H) and H&E staining (I) of lung tissues from PBS and PEG_2000_‐SiNcTI‐Ph/CpG‐ZIF‐8@CM+L groups; The white arrows represent pulmonary nodules. Scale bar, 100 µm. J) Immunofluorescence analysis of intratumoral infiltration of central memory T cells (CD62L^+^CD44^+^) in CT26 tumor‐bearing mice. Scale bar, 100 µm. Blue: Cell nuclei were stained by DAPI. Red: CD62L was labeled by Cy3. Green: CD44 was labeled by FITC. Statistical analysis was calculated via one‐way ANOVA. ^*^ *p* <0.05, ^***^ *p* <0.001, and ^****^ *p* <0.0001. “ns” represents no significant difference. Data are presented as mean ± S.D. (n = 6 biologically independent experiments per group). (1: PBS, 2: PEG_2000_‐SiNcTI‐Ph/CpG‐ZIF‐8@CM group, 3: PEG_2000_‐SiNcTI‐Ph+L group, 4: PEG_2000_‐SiNcTI‐Ph‐ZIF‐8+L group, 5: PEG_2000_‐SiNcTI‐Ph/CpG‐ZIF‐8+L group, 6: PEG_2000_‐SiNcTI‐Ph/CpG‐ZIF‐8@CM+L group; “L” represents laser irradiation.).

PEG_2000_‐SiNcTI‐Ph/CpG‐ZIF‐8@CM‐based tumor environment‐activated PDT was supposed to induce ICD for anti‐tumor immune response in vivo, which should reshape the immune environment beneficial for the anti‐tumor outcome. To decipher this, serum samples, spleens, and tumor tissues of above mice from different groups were collected and analyzed. First, the levels of CD80 and CD86 were profiled to evaluate the maturation of DCs. As shown in **Figure** **5**A, the proportions of mature DCs within spleens from the PEG_2000_‐SiNcTI‐Ph+L, PEG_2000_‐SiNcTI‐Ph‐ZIF‐8+L, and PEG_2000_‐SiNcTI‐Ph/CpG‐ZIF‐8+L groups increased from 14.5% to 17.9%, while that from the PEG_2000_‐SiNcTI‐Ph/CpG‐ZIF‐8@CM+L group reached 20.4%. Mature DCs would engulf DAMPs generated by tumor environment‐activated PDT‐induced ICD and present them to T cells for activation. Supported by immunofluorescence staining (Figure 5B), all laser irradiation groups showed different extents of exposure of CRT as reflected by the red fluorescence, with the strongest fluorescence appearing for the PEG_2000_‐SiNcTI‐Ph/CpG‐ZIF‐8@CM+L group. As a result, analysis of activated T cell populations (i.e., CD4^+^/CD8^+^ T cells) in spleens also revealed that the PEG_2000_‐SiNcTI‐Ph/CpG‐ZIF‐8@CM+L group demonstrated the highest percentage of activated T cells, approximating more than twofold higher than the PBS group (Figure 5C). Correspondingly, the infiltration of CD4^+^ and CD8^+^ T cells within primary tumors was also significantly enhanced for the PEG_2000_‐SiNcTI‐Ph/CpG‐ZIF‐8@CM+L group, guaranteeing the premise of immunotherapy of colon cancer (Figure 5D). As another main reason for the immunosuppressive attribute, the dominated M2 type of macrophage within the tumor severely affects the immunotherapeutic outcome.^[^ ^49^ ^]^ Intriguingly, it was found that the PEG_2000_‐SiNcTI‐Ph/CpG‐ZIF‐8@CM+L treatment effectively repolarized M2‐type macrophages to M1‐type macrophages (Figure 5E). Meanwhile, flow cytometry analysis also confirmed that PEG_2000_‐SiNcTI‐Ph/CpG‐ZIF‐8@CM+L group effectively induced repolarization of M2 macrophage to M1 phenotype^[^ ^50^ ^]^ (Figure S42, Supporting Information). Afterward, the secretion of cytokines in serum from different treatment groups were evaluated by ELISA. Consistently, the concentrations of cytokines positively regulating immune response (i.e., TNF‐α and IFN‐γ) increased for all mice with different material treatments compared with those treated with PBS, with the PEG_2000_‐SiNcTI‐Ph/CpG‐ZIF‐8@CM+L treatment still demonstrating the strongest promotion of generation of TNF‐α and IFN‐γ, leading to approximately sevenfold and approximately threefold enhancements, respectively (Figure 5F). In sum, PEG_2000_‐SiNcTI‐Ph/CpG‐ZIF‐8@CM‐based tumor environment‐activated PDT elicited PD‐L1 blockade‐empowered anti‐tumor immune response to further advance the combat of primary and distant CT26 tumors.

In most cases, tumor metastasis and recurrence will result in the ultimate failure of cancer therapy. Promoted by the positive modulation of immune environment as characterized above, we wondered whether PEG_2000_‐SiNcTI‐Ph/CpG‐ZIF‐8@CM‐based tumor environment‐activated PDT can induce immune memory to inhibit tumor metastasis and recurrence. To evaluate this, CT26 bilateral tumor mice were randomly divided into two groups, which were intravenously injected with PBS and PEG_2000_‐SiNcTI‐Ph/CpG‐ZIF‐8@CM (denoted as the PBS group and PEG_2000_‐SiNcTI‐Ph/CpG‐ZIF‐8@CM+L group; “L” represented laser irradiation.), respectively, where primary tumors were irradiated with an 808 nm laser for 5 min at 24 h post‐injection, followed by re‐challenging the mice with CT26 tumor cells through tail vein after 20 days' treatment to simulate tumor metastasis and recurrence (Figure 5G). Ten days later, the mice were dissected and their lungs were photographed and observed. The results showed that lung tissues of mice in the PBS group exhibited obvious pulmonary nodules, which was in a striking contrast to the effect of the PEG_2000_‐SiNcTI‐Ph/CpG‐ZIF‐8@CM+L group on the inhibition of pulmonary nodule formation (Figure 5H). H&E staining (Figure 5I) further evidenced new metastatic nodules in the lungs of mice in the PBS group, while no obvious tumor metastasis to lung was observed in the PEG_2000_‐SiNcTI‐Ph/CpG‐ZIF‐8@CM+L group. According to immunofluorescence analysis, higher intratumoral infiltration of the central memory CD44^+^CD62L^+^ T cells and CD4^+^/CD8^+^ T cells were observed in the PEG_2000_‐SiNcTI‐Ph/CpG‐ZIF‐8@CM+L group compared with the PBS group (Figure 5J; Figure S43, Supporting Information). This indicated that CD44^+^/CD62L^+^ memory T cells induced by immune response could rapidly transform into tumor antigen‐specific CD4^+^ and CD8^+^ T cells, respectively, once they were attacked by homologous tumor cells again to achieve long‐term protection, thus effectively inhibiting tumor metastasis and recurrence.

## 3 Conclusion

In sum, a tumor environment‐activated photosensitized biomimetic nanoplatform (PEG_2000_‐SiNcTI‐Ph/CpG‐ZIF‐8@CM) based on our developed molecular PS PEG_2000_‐SiNcTI‐Ph was rationally fabricated for the treatment of CT26 colon cancer in mice. This nanoplatform demonstrated advances of tumor environment (i.e., low pH plus high concentrations of ATP)‐activated photodynamic effect with long‐wavelength photon sensitization, evasion of immune surveillance, tumor targeting, and elicitation of effective anti‐tumor immune response. With all advances, PEG_2000_‐SiNcTI‐Ph/CpG‐ZIF‐8@CM realized precise and efficient photodynamic immunotherapy of colon cancer in mice. Meanwhile, PEG_2000_‐SiNcTI‐Ph/CpG‐ZIF‐8@CM‐based tumor environment‐activated PDT could reshape the immune environment in CT26 tumor‐bearing mice by promoting the repolarization of macrophage toward M1 type, increasing intratumoral infiltration of CD4^+^/CD8^+^ T cells, and facilitating the secretion of immunostimulatory cytokines to benefit the photodynamic immunotherapy process. Moreover, the photodynamic immunotherapy induced by PEG_2000_‐SiNcTI‐Ph/CpG‐ZIF‐8@CM generated an immune memory to inhibit the tumor recurrence and metastasis for long‐term protection. The strategy we developed here would open an avenue to rationally design nanoplatforms meeting the concept of precise therapy of cancer.

## 4 Experimental Section

### Materials

2‐methylimidazole (2‐MiM, 98%), HEPES, and ATP trisodium salt solution (≥99%, 100 mM) were obtained from Aladdin Industrial Corporation (Shanghai, China). Zinc nitrate hexahydrate (AR) was obtained from XiLong Scientific Co., Ltd. (Guangdong, China). CpG oligodeoxynucleotide and DPBF were obtained from MedChemExpress (Shanghai, China). Protease Inhibitor Cocktail (Ethylene Diamine Tetraacetic Acid (EDTA)‐free, 100 ×) and EDTA (0.5 M) were obtained from Thermo Fisher Scientific (Shanghai, China). DCFH‐DA was obtained from Maokang Biotechnology Co., Ltd. (Shanghai, China). PD‐1 plasmid (PD‐1 cDNA ORF Clone, Mouse, C‐GFPSpark tag) was obtained from Sino Biological, Inc. (Beijing, China). Dulbecco′s Modified Eagle Medium (DMEM), Roswell Park Memorial Institute (RPMI) 1640, and Fetal Bovine Serum (FBS) were obtained from VivaCell Biosciences (Shanghai, China). Lyso‐Tracker and Mito‐Tracker were obtained from Beyotime Biotechnology (Shanghai, China). Hoechst 33 342 staining solution (1 mg mL^−1^), CCK‐8 Cell Proliferation and Cytotoxicity Assay Kit, MMP Assay Kit (JC‐1), and Calcein‐AM/PI Solution Kit were obtained from Solarbio Life Sciences (Beijing, China). TNF‐α, IFN‐γ, IL‐6, and IL‐10 ELISA kits were obtained from Boster Biological Technology Co., Ltd (Wuhan, China). FITC‐anti‐Mouse CD80 (16‐10A1, 50 µg), PE‐anti‐Mouse CD86 (A17199A, 50 µg), FITC‐anti‐Mouse CD4 (KG1.5, 50 µg), and PE‐anti‐Mouse CD8 (S18018E, 50 µg) antibodies were obtained from Biolegend (Beijing, China).

### Cell Lines and Animals

All the cell lines used in the study were obtained from Procell Life Science & Technology Co., Ltd (Wuhan, China). CT26 cells and the three cell lines (HEK293T, BEAS‐2B, and RAW264.7 cells) were cultured in RPMI 1640 and DMEM medium, respectively, with 10% FBS and 1% penicillin‐streptomycin in a humidified atmosphere at 37 °C containing 5% CO_2._

C57BL/6 mice (male, 5 weeks) were obtained from Gempharmatech Co., Ltd (Jiangsu, China). All mice were raised in the Animal Science Laboratory Center of Nanchang University with a photoperiod of 12 h light/12 h dark, humidity of 40%−68%, and temperature of 22 °C ± 1 °C. All animal experiments were grouped randomly and investigators were blinded to the group allocation during data collection and/or analysis. All animal studies were conducted according to the guidelines approved by Institutional Animal Care and Use Committee (IACUC) of Nanchang University (Nanchang, China, approval number: NCULAE‐20231128009).

### Synthesis of PEG_2000_‐SiNcTI‐Ph

PEG_2000_‐SiNcTI‐Ph was synthesized according to the synthesis route shown in Figure S2 (Supporting Information). Compound **1**‐**8** were synthesized with the reported methods.^[^ ^51^ ^]^

### Synthesis of Compound **9**

A mixture of compound **8** (1 g, 2.49 mmol), sodium methylate (NaOCH_3_, 100 mg, 1.85 mmol), and ammonia (NH_3_, 7 M in methanol (CH_3_OH), 24.9 mmol) were stirred at reflux in CH_3_OH (30 mL) for 1 h. After cooling to room temperature, the mixture was poured into 30 mL of water. The white precipitate was filtered, recrystallized, and dried in a vacuum oven. And compound **9** (692 mg, 65.2%) was obtained as a yellow solid for the next step without further purification. ^1^H NMR (400 MHz, DMSO‐d6) of compound **9**: δ 9.09 (m, 3H), 8.81 (s, 2H), 8.72 (s, 2H), 7.53 (t, 1H), 7.38 (d, 2H), 2.74 (m, 2H), and 1.11 (d, 12H) ppm. ^13^C NMR (101 MHz, DMSO‐d6): δ 167.64, 147.22, 137.10, 130.59, 129.11, 128.62, 127.61, 127.51, 127.21, 124.34, 123.49, 29.17, and 24.12 ppm.

### Synthesis of Compound **10**

Compound **9** (500 mg, 0.018 mol) and 10 mL of quinoline were heated to 210 °C in a flask under argon (Ar_2_), and then silicon tetrachloride (SiCl_4_, 101 mg, 0.60 mmol) was added dropwise. The mixture was stirred for 30 min, the temperature was lowered to 145 °C, then 1 mL of water was added to the reaction mixture and stirred for 1 h. After cooling to room temperature, 60 mL of CH_3_OH was added. The as‐obtained mixture was filtered and purified through a silica gel column with an eluent of dichloromethane (CH_2_Cl_2_). And compound **10** (120 mg, 30.0%) was obtained as a dark green solid. ^1^H NMR (400 MHz, CDCl_3_): δ 10.45 (s, 8H), 9.34 (s, 8H), 7.56 (m, 4H), 7.41 (d, 8H), 2.93 (m, 8H), and 1.30 (d, 48H) ppm. ^13^C NMR (101 MHz, CDCl_3_): δ 167.49, 149.55, 147.06, 136.60, 135.01, 130.47, 129.13, 127.53, 127.06, 126.62, 124.16, 29.56, and 24.11 ppm. HRMS (MALDI‐TOF): Calcd for C_104_H_86_N_12_O_10_Si. Exact Mass: 1690.64, found: 1690.64 (M^+^).

### Synthesis of compound PEG_2000_‐SiNcTI‐Ph

Compound **10** (100 mg, 0.059 mol) and PEG_2000_ (480 mg, 0.24 mol) were dissolved in 10 mL of 1,2‐dichlorobenzene. The mixture was refluxed under Ar_2_ atmosphere for 8 h. After cooling to room temperature, the mixture was purified through a silica gel column (eluent, CH_3_OH:CH_2_Cl_2_ = 1:100). PEG_2000_‐SiNcTI‐Ph was obtained (230 mg, 68%) as a dark green solid. ^1^H NMR (400 MHz, DMSO‐d6): δ 10.61(s, 8H), 9.62 (s, 8H), 7.60 (m, 4H), 7.48 (d, 8H), 3.68 (m, 8H), 3.51 (m), 3.24 (s, 6H), and 1.23 (d, 48H) ppm. MS (MALDI‐TOF): C_106_H_90_N_12_O_10_Si(C_2_H_4_O) _n_ found: 4648.31‐5876.97 (M^+^).

### ROS generation ability of PEG_2000_‐SiNcTI‐Ph

In the study, two fluorescent probes DCFH‐DA and DPBF were used to detect the ROS generation. DCFH was first obtained by adding DCFH‐DA to an aqueous sodium hydroxide (NaOH) solution and stirring for 30 min away from light. The DCFH solution (10 µm, 100 µL) was then added into PEG_2000_‐SiNcTI‐Ph solution (10 µg mL^−1^, 1 mL) and mixed well. Then, the spectra of the mixed solutions were measured by a fluorescence spectrometer. Subsequently, an 808 nm laser with an optical density of 0.3 W cm^−2^ was irradiated for 10 s and the spectrum of the mixed solution was measured. This experiment was performed in parallel for six times and the peak intensity was observed at 525 nm.

For DPBF detection, the DPBF solution (1 mm, 100 µL) was added into PEG_2000_‐SiNcTI‐Ph solution (30 µg mL^−1^, 1 mL) and mixed well. Then, the spectrum of the mixed solution was measured by a UV–vis spectrophotometer. Similarly, the 808 nm laser with a density of 0.3 W cm^−2^ was irradiated for 10 s and the spectrum of the mixed solution was measured. This experiment was performed in parallel for six times and the peak intensity was observed at 410 nm.

### Synthesis of PEG_2000_‐SiNcTI‐Ph‐ZIF‐8 and PEG_2000_‐SiNcTI‐Ph/CpG‐ZIF‐8

For the preparation of PEG_2000_‐SiNcTI‐Ph‐ZIF‐8, first, 40 mg of Zn (NO_3_)_2_·6H_2_O and 0.77 g of 2‐MiM were dissolved in 1 mL and 4 mL of deionized (DI) water, respectively. After that, 8 mg of PEG_2000_‐SiNcTI‐Ph was added to the Zn (NO_3_)_2_ solution and mixed for 2 min, followed by adding the 2‐MiM solution and magnetically stirring for 5 min. The light green product was obtained by centrifugation at 12 000 rpm min^−1^ for 10 min and washed three times with DI water.

For the synthesis of PEG_2000_‐SiNcTI‐Ph/CpG‐ZIF‐8, similarly, 8 mg of PEG_2000_‐SiNcTI‐Ph and 500 µg of CpG was added to above Zn (NO_3_)_2_·6H_2_O solution and mixed for 2 min, followed by adding the 2‐MiM solution and magnetically stirring for 5 min. The light green product was obtained by centrifugation at 12 000 rpm min^−1^ for 10 min and washed three times with DI water.

### Preparation of HM, RM, and PEG_2000_‐SiNcTI‐Ph/CpG‐ZIF‐8@CM

For extraction of HM, HEK293T‐PD‐1 cells were first obtained by transfecting PD‐1 plasmid (C‐GFPSpark tag) into HEK293T cells and then centrifuged for 3 min at 300 g and rinsed three times with PBS. The cells were then resuspended in cell membrane isolation buffer (0.25 mM sucrose, 1 mM EDTA, 20 mM HEPES‐NaOH, and protease inhibitor; pH = 7.4) and crushed with an ultrasonic pulverizer for 20 s. The supernatant was then collected and centrifuged again at 10 000 g for 20 min to discard the pellet. The cell membrane was collected by re‐centrifugation at 30 000 g for 60 min, which were finally freeze‐dried and stored in at −80 °C. The above process was always performed at 4 °C.

RM was extracted according to the reported method.^[^ ^32^ ^]^ First, the obtained RBCs from mice were resuspended in 0.25 × PBS for 2 h to induce RBCs rupture. Then, RM was collected by centrifugation at 14 000 g for 30 min. The above procedure was repeated until the RM solution was colorless. All above experimental operations were carried out at 4 °C.

Finally, 1 mL of PEG_2000_‐SiNcTI‐Ph/CpG‐ZIF‐8 (0.5 mg mL^−1^) and 1 mL of mixed cell membrane fragments (0.5 mg mL^−1^, HM:RM = 1:1) were mixed in PBS solution. Then, the mixture was sonicated for 30 s and sequentially passed through 800 nm and 400 nm porous polycarbonate membranes by an Avanti mini extruder to obtain the PEG_2000_‐SiNcTI‐Ph/CpG‐ZIF‐8@CM.

### Release of PEG_2000_‐SiNcTI‐Ph

To study pH‐ and ATP‐responsive release of PEG_2000_‐SiNcTI‐Ph, PEG_2000_‐SiNcTI‐Ph/CpG‐ZIF‐8@CM was dispersed in PBS (pH 7.4 or 5.5) and/or incubated with different concentrations of ATP (0, 2, and 5 mm), respectively. At different time periods, the supernatant was obtained by centrifugation and the absorbance at 790 nm was measured with a UV–vis spectrophotometer. The released profiles of PEG_2000_‐SiNcTI‐Ph at each time point were calculated according to the standard curves.

### Flow Cytometry

To evaluate the expression of PD‐1 protein on HEK293‐PD‐1 cells and CD47 protein on RBC, HEK293‐PD‐1 cells and RBCs were washed with PBS for three times, and then incubated with FITC‐anti‐PD‐1 and FITC‐anti‐CD47 for 30 min, respectively, and finally washed with PBS for three times for flow cytometry analysis.

### Sodium Dodecyl Sulfate‐Polyacrylamide Gel Electrophoresis (SDS‐PAGE) Analysis

Samples were added to 10% SDS‐PAGE gels and separation was initiated under 120 V. After the separation, incubate the gel for staining by adding it to the coomassie blue staining solution. After that, it was washed with coomassie blue staining destaining solution. Finally, wash the gel with water.

### Western Blotting

Samples were added to 10% SDS‐PAGE gels for separation, followed by being transferred to a polyvinylidene fluoride (PVDF) membrane and blocked with 5% skimmed milk for 1 h at room temperature. After incubation with the corresponding primary antibodies, the membrane was washed three times with TBST for 10 min and then incubated with the secondary antibody for 1 h at room temperature. The membrane was washed three times with TBST and then stained with enhanced chemiluminescent (ECL) detection reagent. Proteins were observed using a digital gel image analysis.

### Lysosomal/Mitochondrial Localization Experiment

CT26 cells were seeded into the cell culture dishes for 24 h of incubation at 37 °C. Afterward, fresh medium containing Cy5‐labeled PEG_2000_‐SiNcTI‐Ph/CpG‐ZIF‐8@CM was added for another 2 h of incubation. After washing the cells with PBS for three times, they were stained with Lyso Tracker/Mito Tracker for 20 min at 37 °C. Finally, the cells were washed with PBS for three times and then imaged by CLSM.

### Intracellular ROS Generation

CT26 cells or BEAS‐2B cells were seeded into the cell culture dishes for 24 h of incubation at 37 °C. Fresh medium containing PEG_2000_‐SiNcTI‐Ph/CpG‐ZIF‐8@CM (PEG_2000_‐SiNcTI‐Ph equivalent dose: 30 µg mL^−1^) was added for another 4 h of incubation. After washing the cells with PBS for three times, they were incubated with 10 µm DCFH‐DA for 20 min at 37 °C in the dark. After washing, the cells were irradiated with an 808 nm laser (1 W cm^−2^) for 15 min, followed by CLSM imaging. Conditions: excitation wavelength: 488 nm; emission filter: 500–550 nm.

### Quantum Chemical Calculations

The ground‐state (S_0_) geometries of monomer and aggregation dimer models were optimized using density functional theory (DFT) at the B3LYP‐D3 (BJ)^[^ ^52^, ^53^, ^54^ ^]^ /6‐31G (d) level. Both the singlet and triplet excitation energies were calculated at the time‐dependent (TD) DFT/6‐31G (d) level with the polarizable‐continuum model in water, with three selected B3LYP, M062X, and *ω*B97XD functionals. And the TDDFT calculations suggested that the (TD)‐B3LYP/6‐31G (d) level was proved to reasonably reproduce the experimental measurement and was employed in this work (Table S1, Supporting Information). The corresponding HOMOs and LUMOs for ground states, as well as the hole and electron distribution for various excited states, were plotted and rendered using variational mode decomposition (VMD) code.^[^ ^55^ ^]^ All the DFT and TDDFT calculations were performed using the Gaussian 16 program.^[^ ^56^ ^]^ The SOC elements between singlet and triplet states were evaluated using the linear‐response (LR) TDDFT method employing Casida‐type wave functions and the Breit‐Pauli (BP) spin‐orbit Hamiltonian with an effective charge approximation by PySOC code.^[^ ^57^ ^]^

### Endocytosis Experiments

CT26 cells or RAW264.7 cells were seeded into 24‐well plates with a density of 1 × 10^5^ cells well^−1^ for 24 h of incubation. Different samples were added for another 2 h of incubation. Then, the medium was removed and washed with PBS for three times, followed by staining with Hoechst 33 342 for 15 min. After that, the samples were washed with PBS and imaged by a fluorescence microscope to investigate the cell endocytosis of PEG_2000_‐SiNcTI‐Ph/CpG‐ZIF‐8 or PEG_2000_‐SiNcTI‐Ph/CpG‐ZIF‐8@CM.

### Cell Viability Test

CT26 cells or BEAS‐2B cells were seeded into 96‐well plates with a density of 5 × 10^3^ cells well^−1^ for 12 h of incubation. Fresh medium containing different materials (equivalent contents of PEG_2000_‐SiNcTI‐Ph in different materials) was added for another 12 h of incubation and the medium was removed and washed with PBS for three times. Afterward, the cells were irradiated with an 808 nm laser (1 W cm^−2^) for 2 min. Next, the old culture medium was replaced with serum‐free medium containing 10% CCK8 for 2 h of incubation at 37 °C. The absorbance at 450 nm was measured by a microplate reader.

### Cellular Mitochondrial Damage by JC‐1 Assays

CT26 cells or BEAS‐2B cells were seeded into 12‐well plates for 12 h of incubation. Fresh medium containing different materials (equivalent dose of PEG_2000_‐SiNcTI‐Ph: 30 µg mL^−1^) was added for 4 h of incubation. After that, the cells were irradiated with an 808 nm laser (1 W cm^−2^) for 15 min. Afterward, the medium was removed and the cells were stained with 10 µg mL^−1^ of JC‐1 for 20 min at 37 °C. Finally, the cells were washed with PBS for three times and then imaged by a fluorescence microscope.

### Live/Dead Cell Staining

CT26 cells or BEAS‐2B cells were seeded into 24‐well plates at a density of 1 × 10^5^ cells well^−1^ for 24 h of incubation. Afterward, fresh medium containing different materials (equivalent dose of PEG_2000_‐SiNcTI‐Ph: 30 µg mL^−1^) was added for 4 h of incubation. Subsequently, the cells were washed and irradiated with an 808 nm laser (1 W cm^−2^) for 15 min. After that, the cells were incubated at 37 °C for another 4 h, then successively stained with Calcein‐AM and PI. Subsequently, the cells were washed with PBS for three times and observed by a fluorescence microscope.

### Analysis of DAMPs

CT26 cells were seeded into 6‐well plates for 24 h. Fresh culture media containing PBS or PEG_2000_‐SiNcTI‐Ph/CpG‐ZIF‐8@CM (equivalent dose of PEG_2000_‐SiNcTI‐Ph: 30 µg mL^−1^) was then added and incubated for 12 h. Subsequently, the cells were washed and irradiated with an 808 nm laser (1 W cm^−2^) for 15 min. Finally, we took apoptotic CT26 cells for WB analysis of CRT and cell supernatant for WB analysis of HMGB‐1 and HSP‐70.

### Evaluation of the Induced Immune Response

CT26 cells (5 × 10^5^ cells well^−1^) were seeded into 6‐well plates for 24 h of incubation. Fresh culture media containing PBS, CpG, PEG_2000_‐SiNcTI‐Ph, PEG_2000_‐SiNcTI‐Ph‐ZIF‐8, PEG_2000_‐SiNcTI‐Ph/CpG‐ZIF‐8, or PEG_2000_‐SiNcTI‐Ph/CpG‐ZIF‐8@CM (equivalent dose of PEG_2000_‐SiNcTI‐Ph: 30 µg mL^−1^) was then added and incubated for 12 h. Then, the supernatant was collected and incubated for 24 h with a density of 3 × 10^6^ mouse spleen immune cells/well. Finally, immune cells were collected and stained for flow cytometric analysis, and supernatants were collected for determination of relevant cytokines by ELISA kits.

### In Vivo Biocompatibility Evaluation

To evaluate the biocompatibility of PEG_2000_‐SiNcTI‐Ph/CpG‐ZIF‐8@CM, PEG_2000_‐SiNcTI‐Ph/CpG‐ZIF‐8@CM (equivalent dose of PEG_2000_‐SiNcTI‐Ph: 2.5 mg kg^−1^ body weight) was injected into the mice via tail vein. Mice were sacrificed at 21 days post injection and major organs were collected for H&E staining. Meanwhile, mouse blood was obtained for routine analysis of liver function, kidney function, and other biochemical indicators.

### In Vivo Blood Retention and Targeting Capability

To evaluate the blood circulation time of PEG_2000_‐SiNcTI‐Ph/CpG‐ZIF‐8@CM, C57BL/6 mice were divided into three groups and administered with PEG_2000_‐SiNcTI‐Ph, PEG_2000_‐SiNcTI‐Ph/CpG‐ZIF‐8, or PEG_2000_‐SiNcTI‐Ph/CpG‐ZIF‐8@CM at a PEG_2000_‐SiNcTI‐Ph equivalent dose of 2.5 mg kg^−1^. At different time points, mouse blood was taken out through the eyeball. The blood samples were then photographed by a fluorescence imaging device. The fluorescence intensity of the blood was analyzed by an image J software to estimate the content of these nanocomposites in blood and calculate their blood half‐life time.

Tumor models were generated by injection of CT26 cells (5 × 10^6^ cells mouse^−1^) on the right side of each mouse. When the tumor volume reached ≈80 mm^3^, the mice were divided into two groups (n = 3) and injected with ICG‐labeled PEG_2000_‐SiNcTI‐Ph/CpG‐ZIF‐8, ICG‐labeled PEG_2000_‐SiNcTI‐Ph/CpG‐ZIF‐8@CM_N_, and ICG‐labeled PEG_2000_‐SiNcTI‐Ph/CpG‐ZIF‐8@CM (equivalent dose of PEG_2000_‐SiNcTI‐Ph: 2.5 mg kg^−1^ body weight) via tail vein, respectively. At different time periods, mice were imaged by a fluorescence imaging system. The fluorescence intensity at the tumor was evaluated by image J software. Finally, the mice were sacrificed at 24 h post injection and their tumors were isolated and photographed by the imaging instrument.

### In Vivo PEG_2000_‐SiNcTI‐Ph/CpG‐ZIF‐8@CM‐Mediated Tumor Suppression

CT26 cells (5 × 10^6^ cells mouse^−1^) were injected subcutaneously on the right side of C57BL/6 mice to generate primary tumors. After the tumor volume reached ≈80 mm^3^, the left side of the same mice was inoculated with the same number of CT26 cells to establish a bilateral tumor model. The mice were randomly divided into six groups (n = 6) (denoted as 1: the PBS group, 2: the PEG_2000_‐SiNcTI‐Ph/CpG‐ZIF‐8@CM group, 3: the PEG_2000_‐SiNcTI‐Ph+L group, 4: the PEG_2000_‐SiNcTI‐Ph‐ZIF‐8+L group, 5: the PEG_2000_‐SiNcTI‐Ph/CpG‐ZIF‐8+L group, 6: the PEG_2000_‐SiNcTI‐Ph/CpG‐ZIF‐8@CM+L group; “L” represented laser irradiation). Different materials were injected into mice via tail vein, followed by irradiation of primary tumors at 24 h post‐injection (808 nm, 1 W cm^−2^, 5 min). Afterward, mouse body weight and bilateral tumor volume were measured every two days. Tumor volume was calculated as (width^2^ × length)/2. In the in vivo efficacy study, tumor growth was tracked until day 20 when the mice were executed and tumor tissue and major organs were then collected for further analysis. And the spleens were made into single‐cell suspensions. Cell suspension was then stained with the FITC‐anti‐mouse CD80 and PE‐anti‐mouse CD86 antibodies to detect DCs, and T cells were detected with the FITC‐anti‐mouse CD4 and PE‐anti‐mouse CD8 antibodies.

To further evaluate the ability of PEG_2000_‐SiNcTI‐Ph/CpG‐ZIF‐8@CM to inhibit tumor metastasis and recurrence, a lung metastasis and tumor recurrence model was established. CT26 cells (5 × 10^6^ cells mouse^−1^) were injected subcutaneously on the right side of C57BL/6 mice to generate primary tumors. After the tumor volume reached ≈80 mm^3^, the distant tumors of mice were generated through inoculating the same number of CT26 cells on the left side of the same mice. The mice were randomly divided into two groups (n = 6) (denoted as 1: the PBS group, 2: the PEG_2000_‐SiNcTI‐Ph/CpG‐ZIF‐8@CM+L group; “L” represented laser irradiation). PBS and PEG_2000_‐SiNcTI‐Ph/CpG‐ZIF‐8@CM were injected into mice via tail vein, followed by irradiation of primary tumors at 24 h post‐injection (808 nm, 1 W cm^−2^, 5 min). After 20 days' of treatment, CT26 cells were injected into the mice again via tail vein to establish a lung metastasis and tumor recurrence model. Ten days later, the mice were dissected and their lungs were observed and further stained with H&E for histological analysis.

### Statistical Analysis

All experiments were performed at least three times and all data represented mean ± s.d. GraphPad Prism 8 was used to conduct the statistical analysis between the test and control groups using the Student's *t*‐test and one‐way ANOVA. ^*^ *p* <0.05, ^**^ *p* <0.01, ^***^ *p* <0.001, and ^****^ *p* <0.0001 represented different statistical significance. “ns” stood for no significant difference.

## Conflict of Interest

The authors declare no conflict of interest.

## Supporting information

Supporting Information

## Data Availability

The data that support the findings of this study are available from the corresponding author upon reasonable request.
