# Supplementary material for: A Tumor Environment‐Activated Photosensitized Biomimetic Nanoplatform for Precise Photodynamic Immunotherapy of Colon Cancer
Source: Adv Sci (Weinh). 2024 May 10;11(28):2402465. doi: 10.1002/advs.202402465 (PMC11267356; doi:10.1002/advs.202402465)
Supplement: Supplementary file 1 — Supporting Information [file ADVS-11-2402465-s001.docx]

Supporting Information

A tumor environment-activated photosensitized biomimetic nanoplatform for precise photodynamic immunotherapy of colon cancer

Mengmeng Xiong, Ying Zhang, Huan Zhang, Qiaoqiao Shao, Qifan Hu, Junjie Ma, Yiqun Wan, Lan Guo *, Xin Wan, Haitao Sun, Zhongyi Yuan* and Hao Wan *

**

**

**Figure S1.** Structure of naphthalocyanine derivatives.

**Figure S2.** Synthetic route of PEG_2000_-SiNcTI-Ph. i) I_2_, Br_2_, 0 °C, 4 h, 74.5%; ii) NBS, AIBN, CCl_4_, *hv*, reflux, 10 h, 69.7%; iii) dimethyl fumarate, KI, DMF, 90 °C, 12 h, 58.0%; iv) KOH, CH_3_OH, H_2_O, reflux, 12 h, 90.0%; v) 240 °C, 3 h, 95%; vi) acetic acid, 2, 6-diisopropylaniline, reflux, 8 h, 76.5%; vii) KI, CuCN, DMF, reflux, 8 h, 68.0%; viii) CH_3_ONa, NH_3_, CH_3_OH, 65 °C, 1 h, 65.2%; ix) SiCl_4_, quinoline, 210 °C, 30 min; H_2_O, 145 °C, 1 h, 30.0%; x) 1,2-dichlorobenzene, mPEG_2000_, reflux, 8 h, 68.0%.


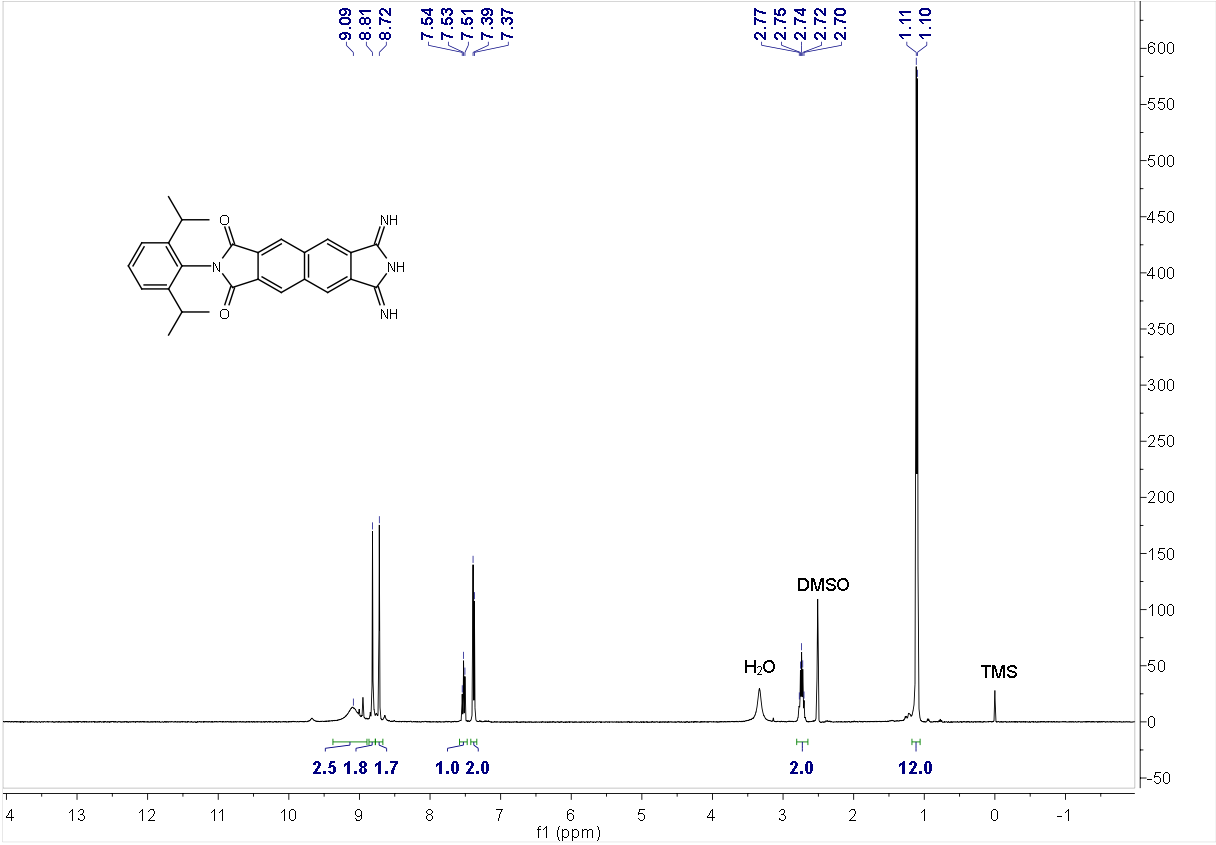


**Figure S3.** ^1^H NMR of compound **9** in DMSO-d6 (400 MHz).


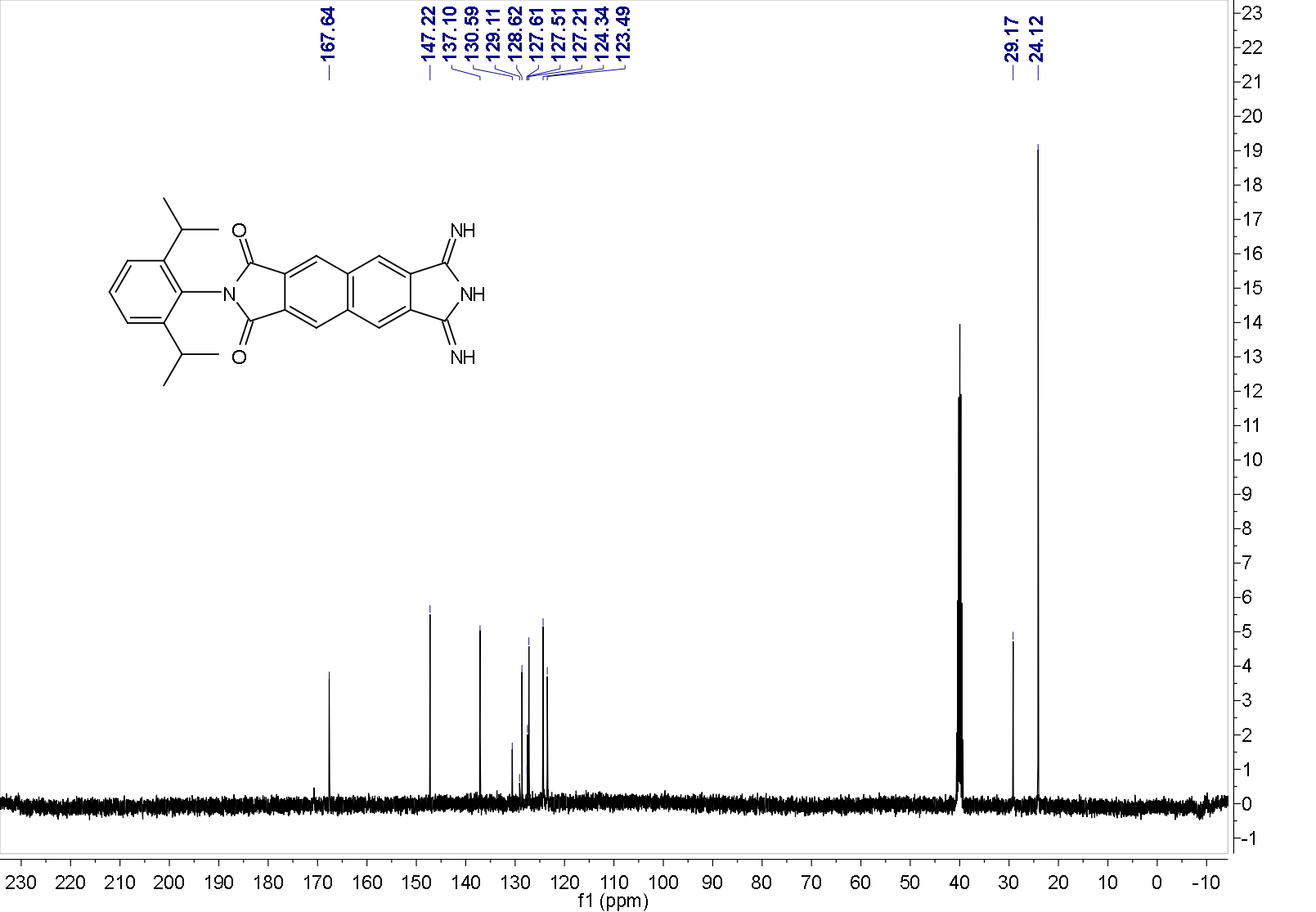


**Figure S4.** ^13^C NMR of compound **9** in DMSO-d6 (101 MHz).


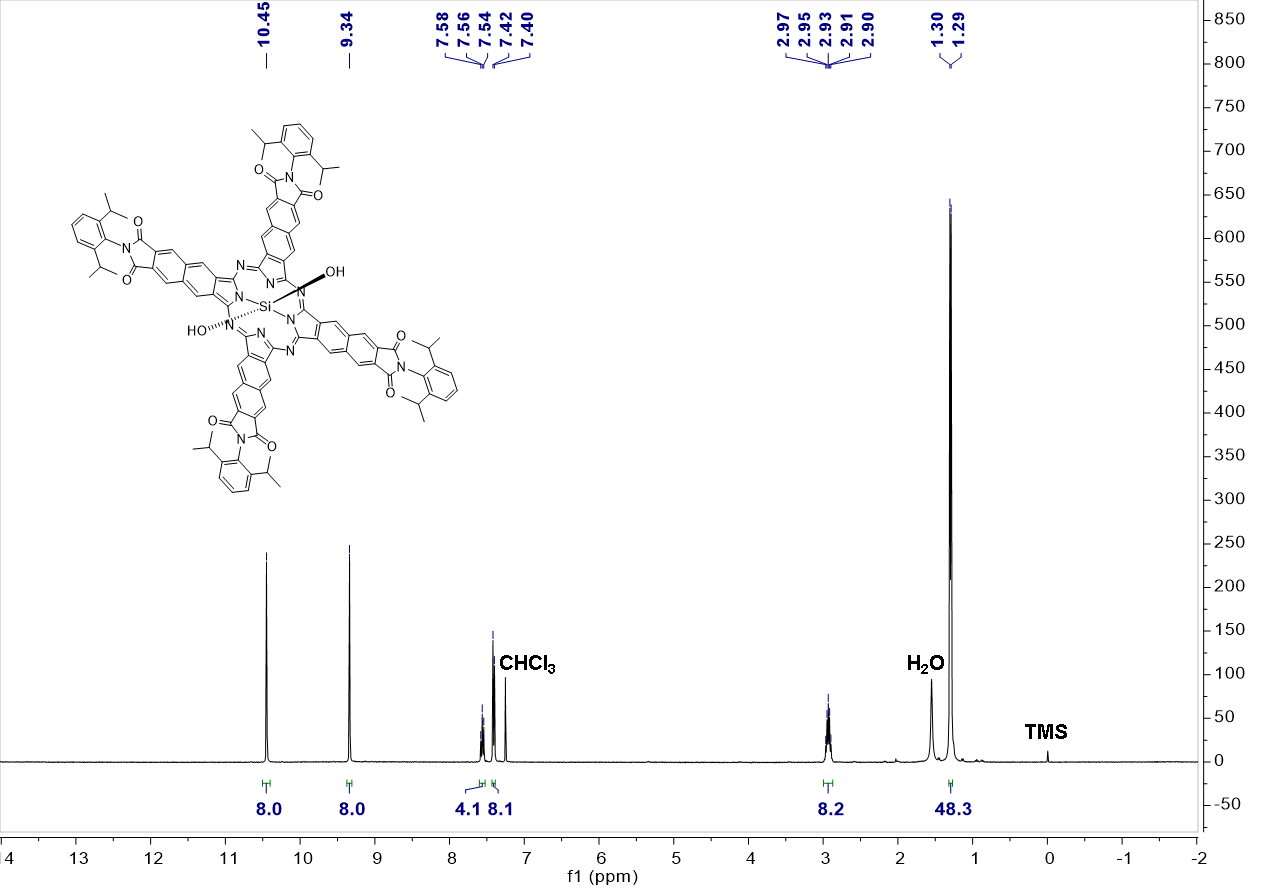


**Figure S5.** ^1^H NMR of compound **10** in CDCl_3_ (400 MHz).


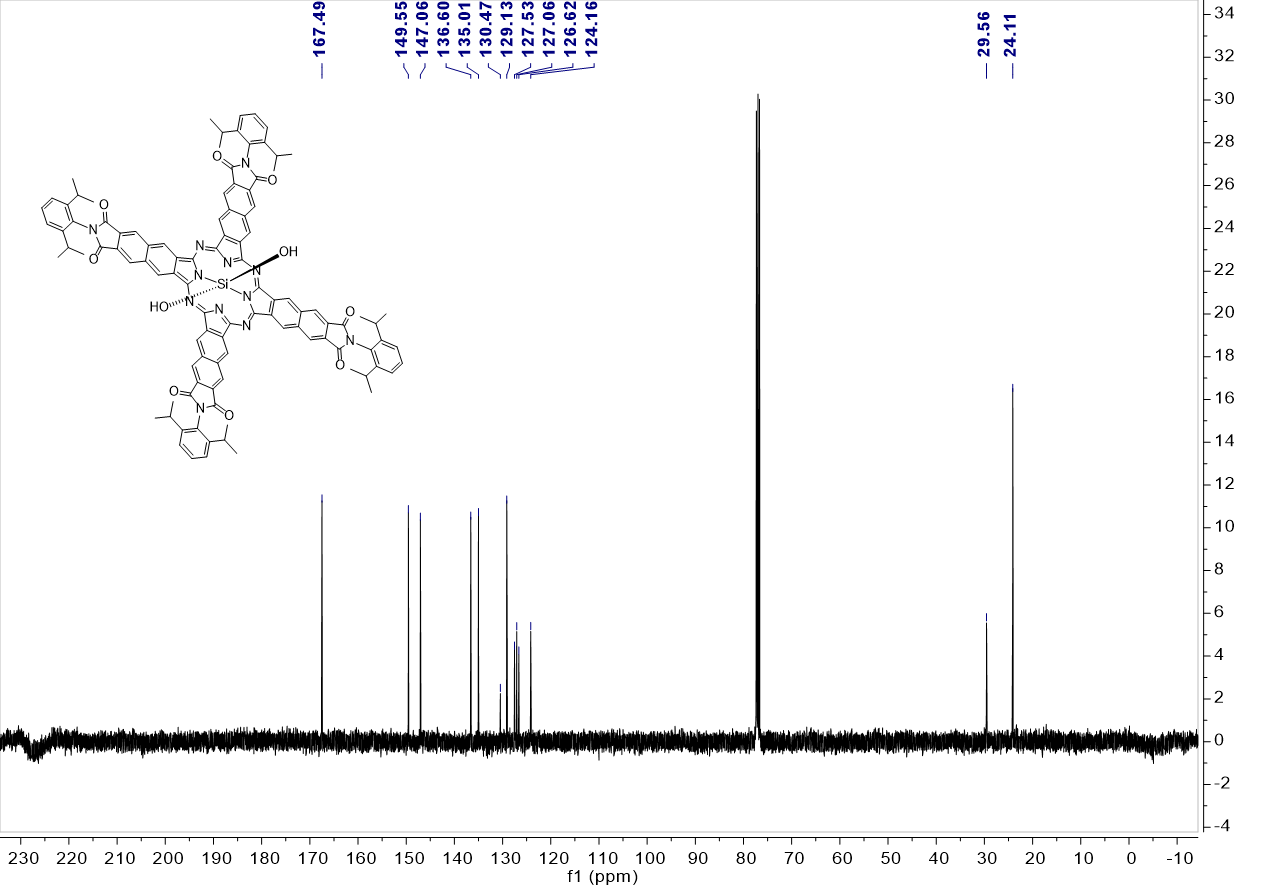


**Figure S6.** ^13^C NMR of compound **10** in CDCl_3_ (101 MHz).


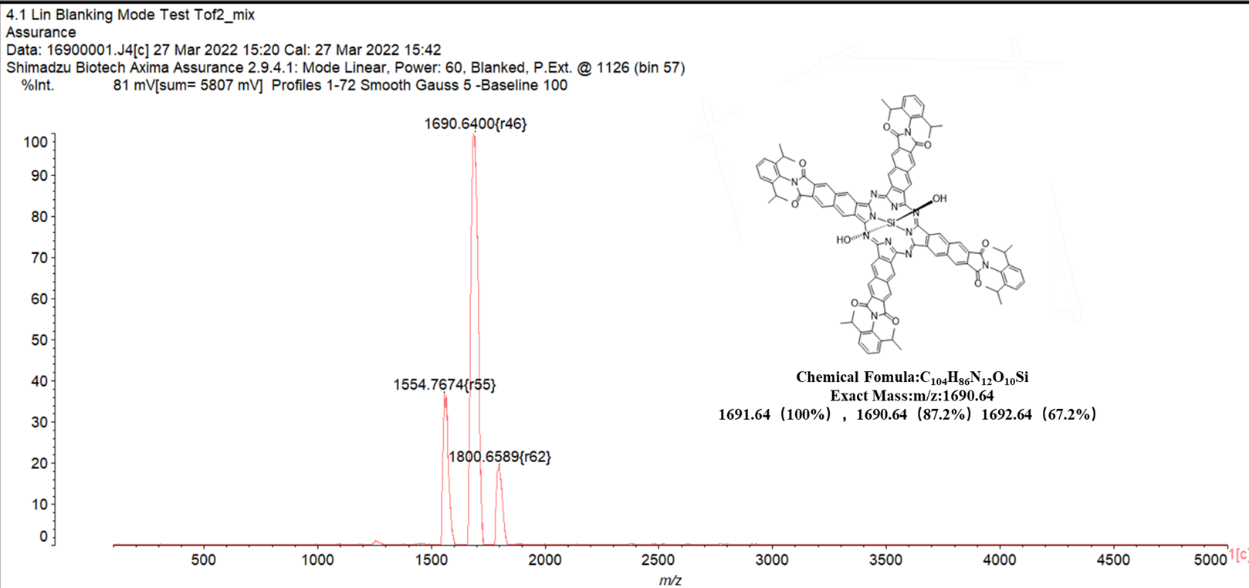


**Figure S7.** MALDI-TOF-MS of compound **10.**

**
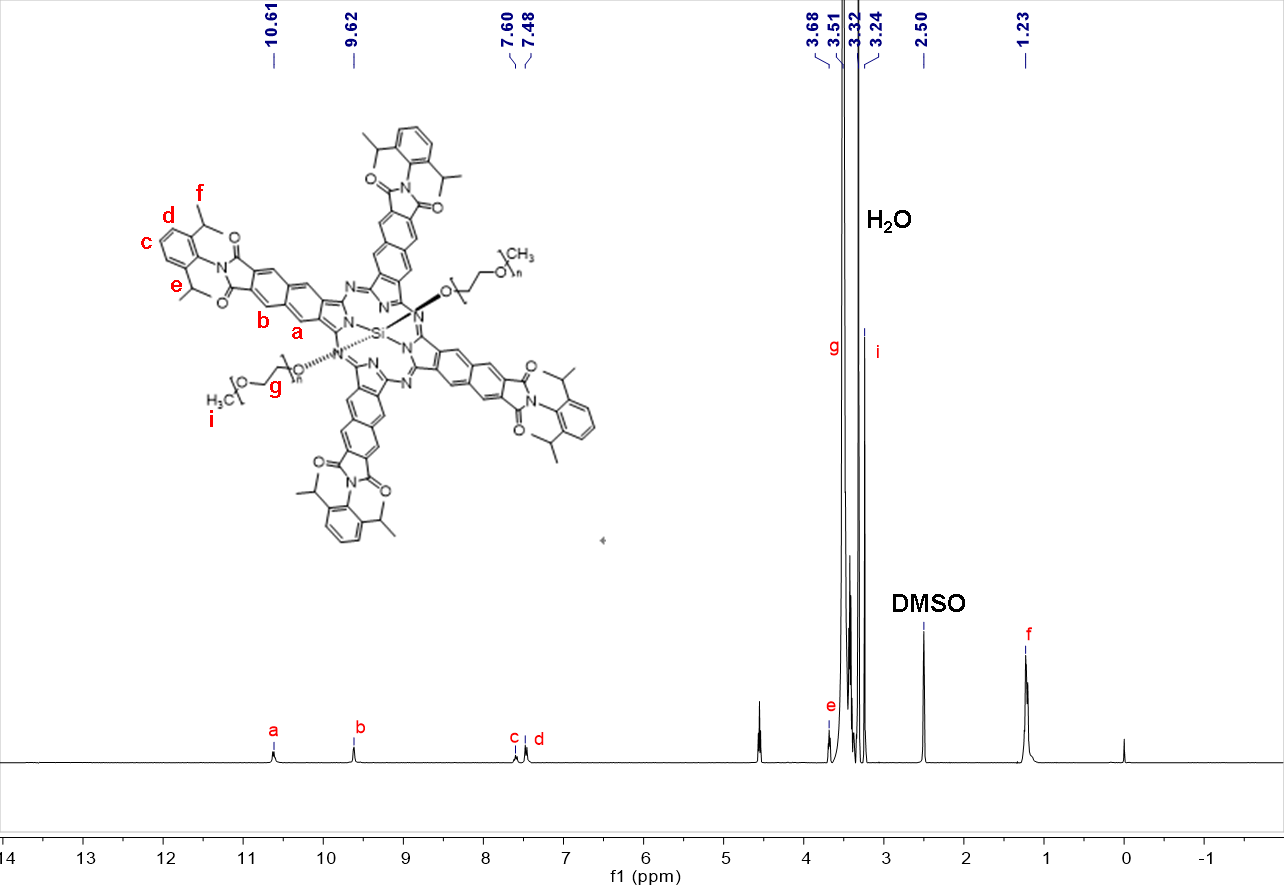
**

**Figure S8.** ^1^H NMR of PEG_2000_-SiNcTI-Ph in DMSO-d6 (400 MHz).


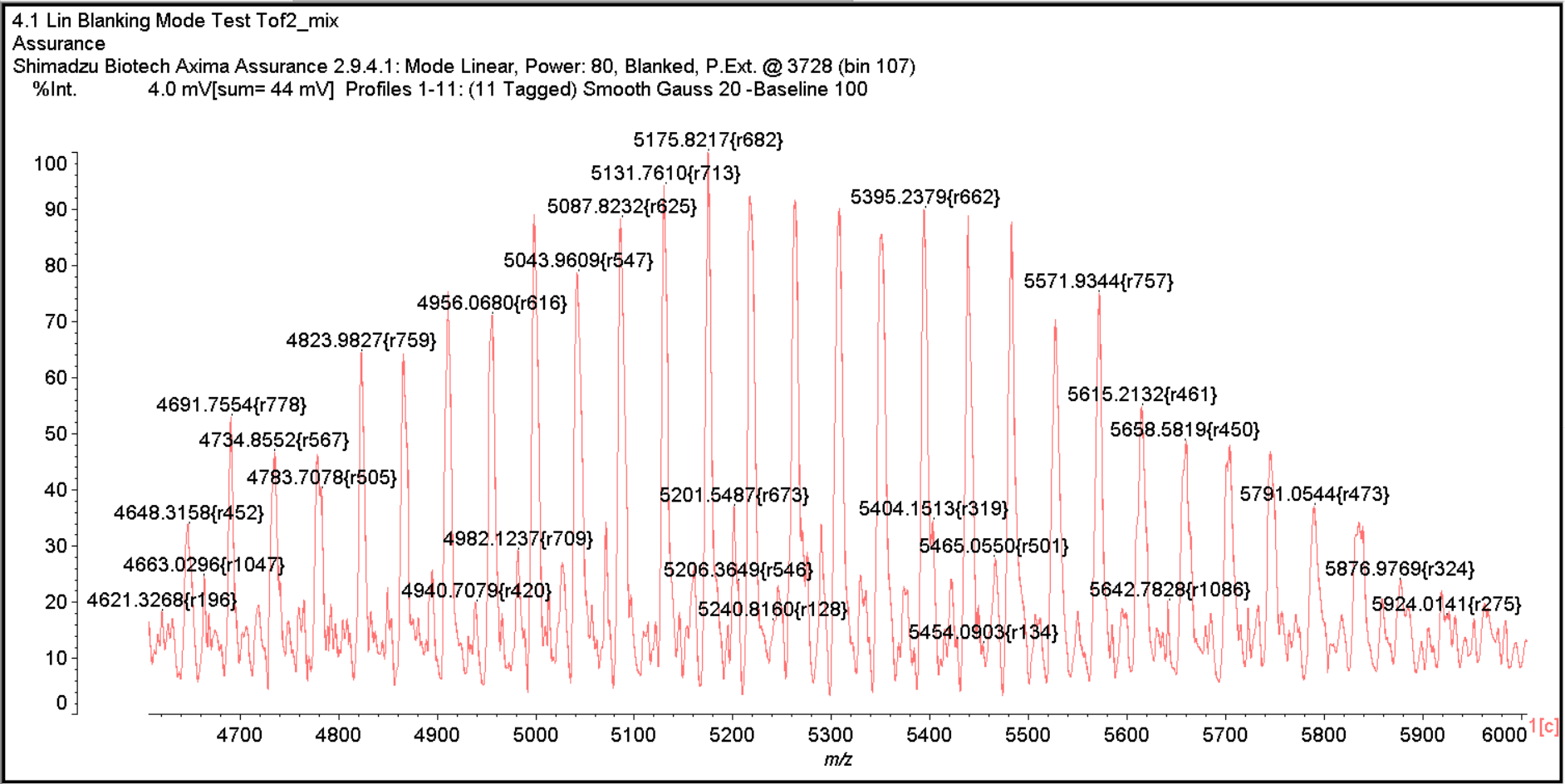


**Figure S9.** MALDI-TOF-MS of PEG_2000_-SiNcTI-Ph.


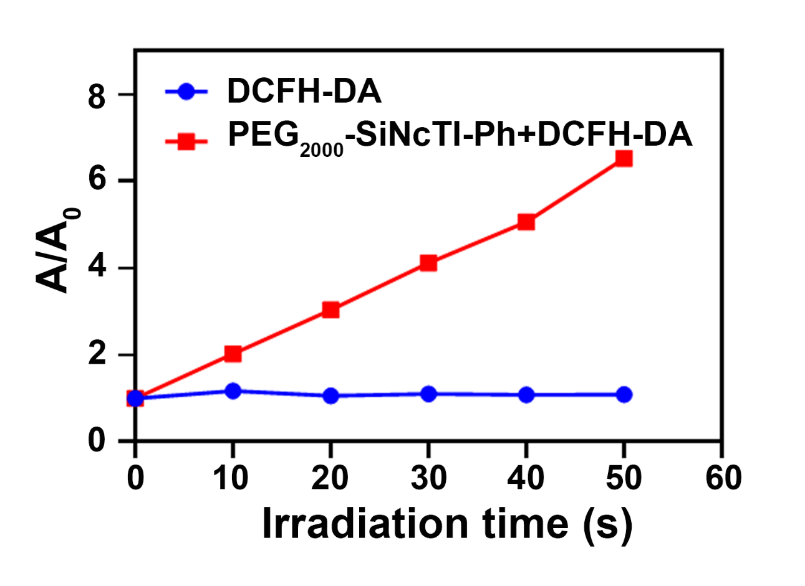


**Figure S10.** ROS generation ability of PEG_2000_-SiNcTI-Ph under 808 nm laser irradiation, measured by DCFH-DA at 525 nm. A/A_0_ represented the ratio of the fluorescence intensity measured at 525 nm to the fluorescence intensity measured at 525 nm without laser irradiation.


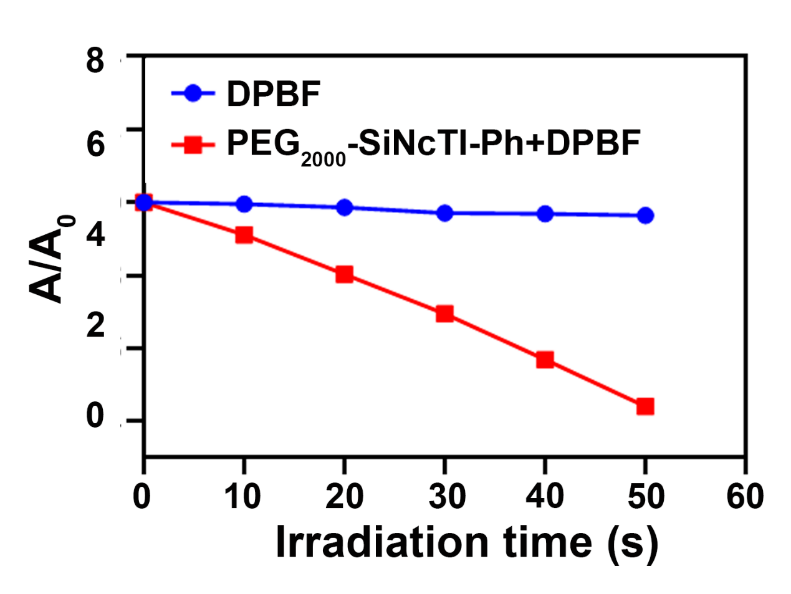


**Figure S11.** ROS generation ability of PEG_2000_-SiNcTI-Ph under 808 nm laser irradiation, measured by DPBF at 410 nm. A/A_0_ represented the ratio of the absorbance measured at 410 nm to the absorbance measured at 410 nm without laser irradiation.


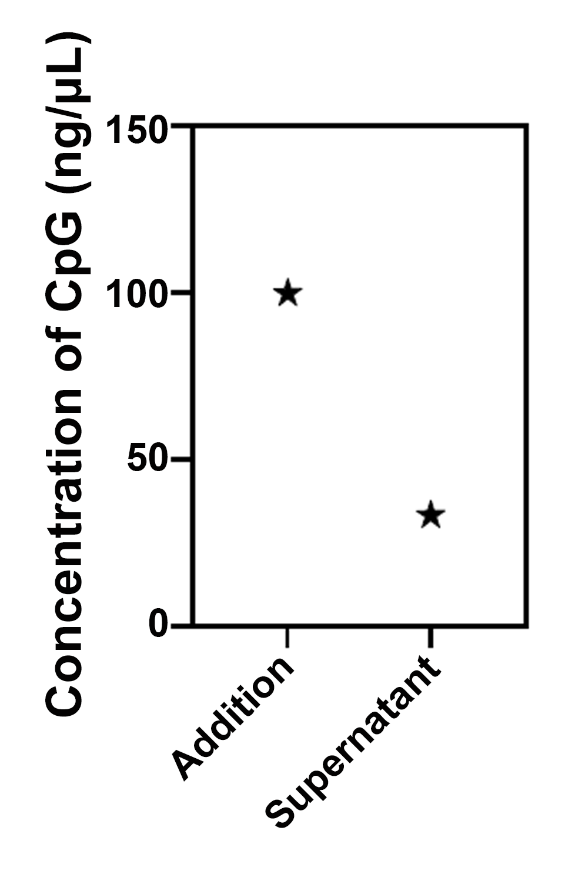


**Figure S12.** Concentration of CpG added and in the supernatant after centrifugation once the encapsulation by ZIF-8 was finished.


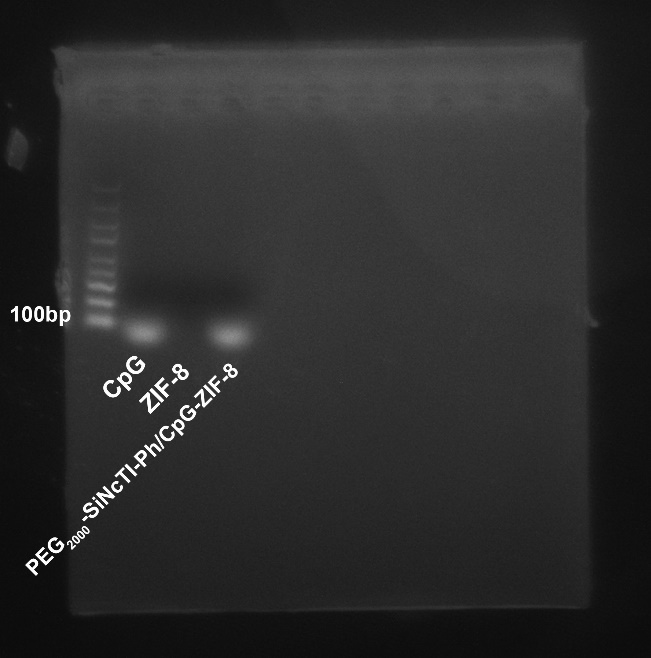


**Figure S13.** Agarose gel electrophoresis of different materials.


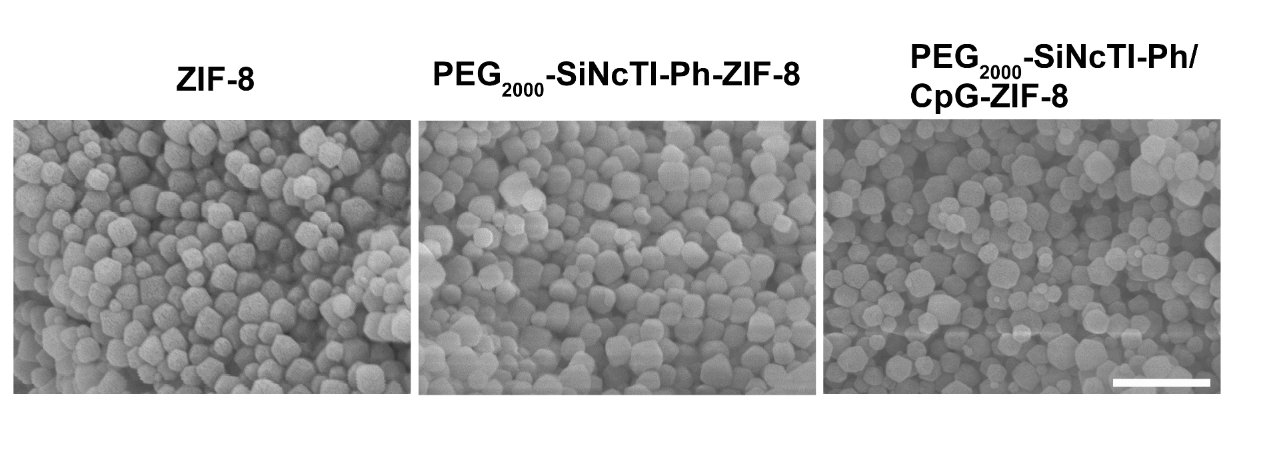


**Figure S14.** SEM images of different NPs. Scale bar, 500 nm.


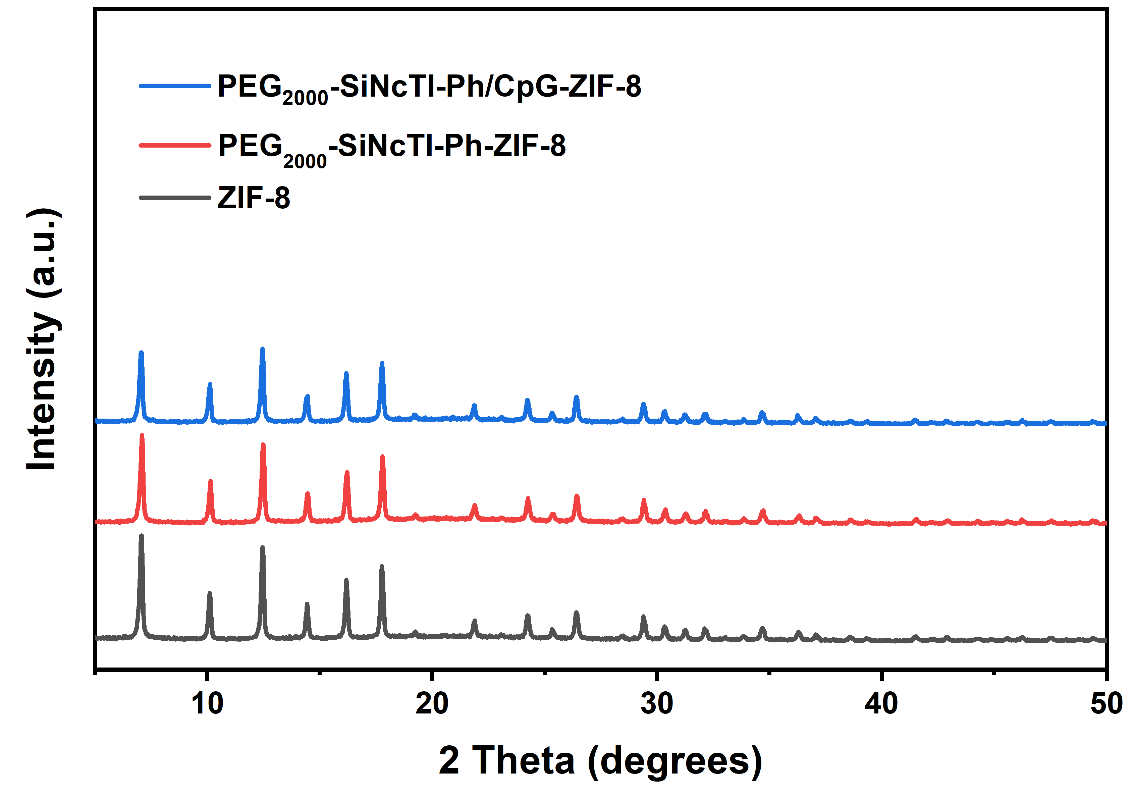


**Figure S15.** XRD patterns of different NPs.


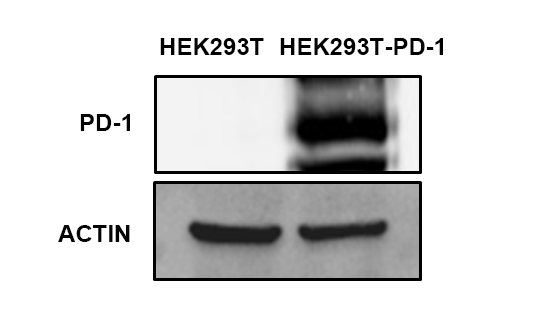


**Figure S16.** WB analysis of PD-1 protein expression on the HEK293T and HEK293T-PD-1 cells.


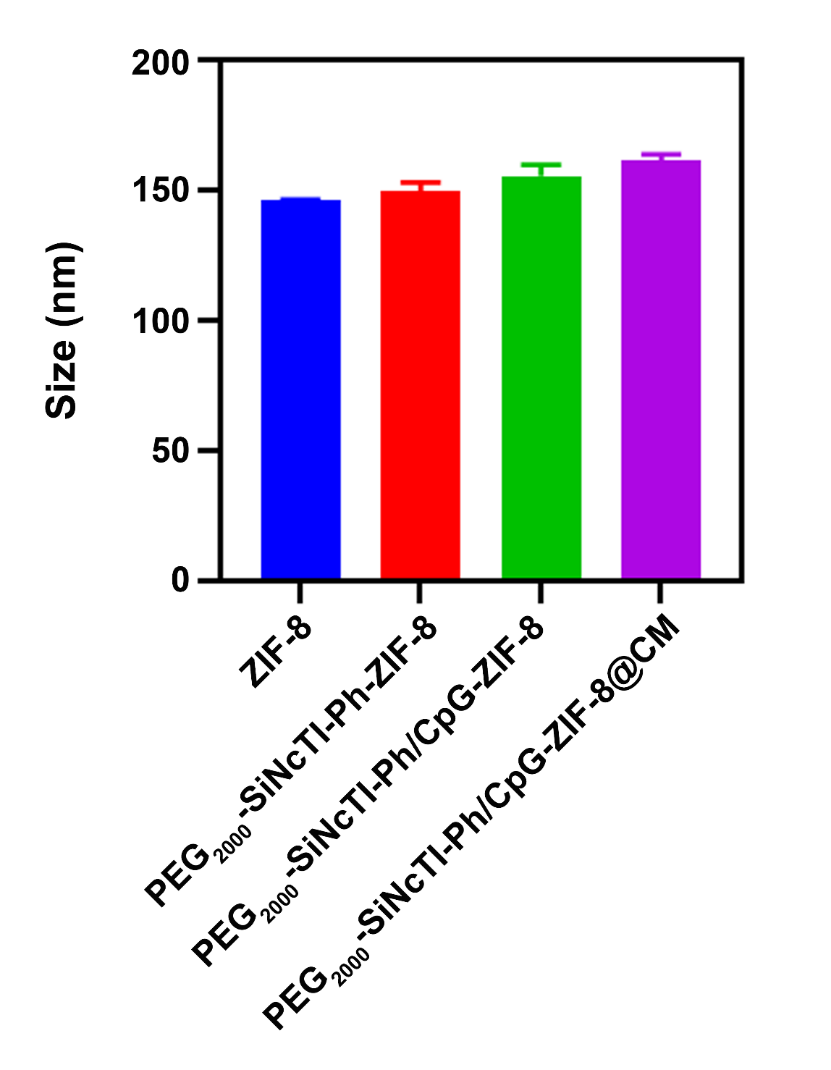


**Figure S17.** Size distribution of different NPs. Data are presented as mean ± S.D. (n = 3 independent experiments per group).


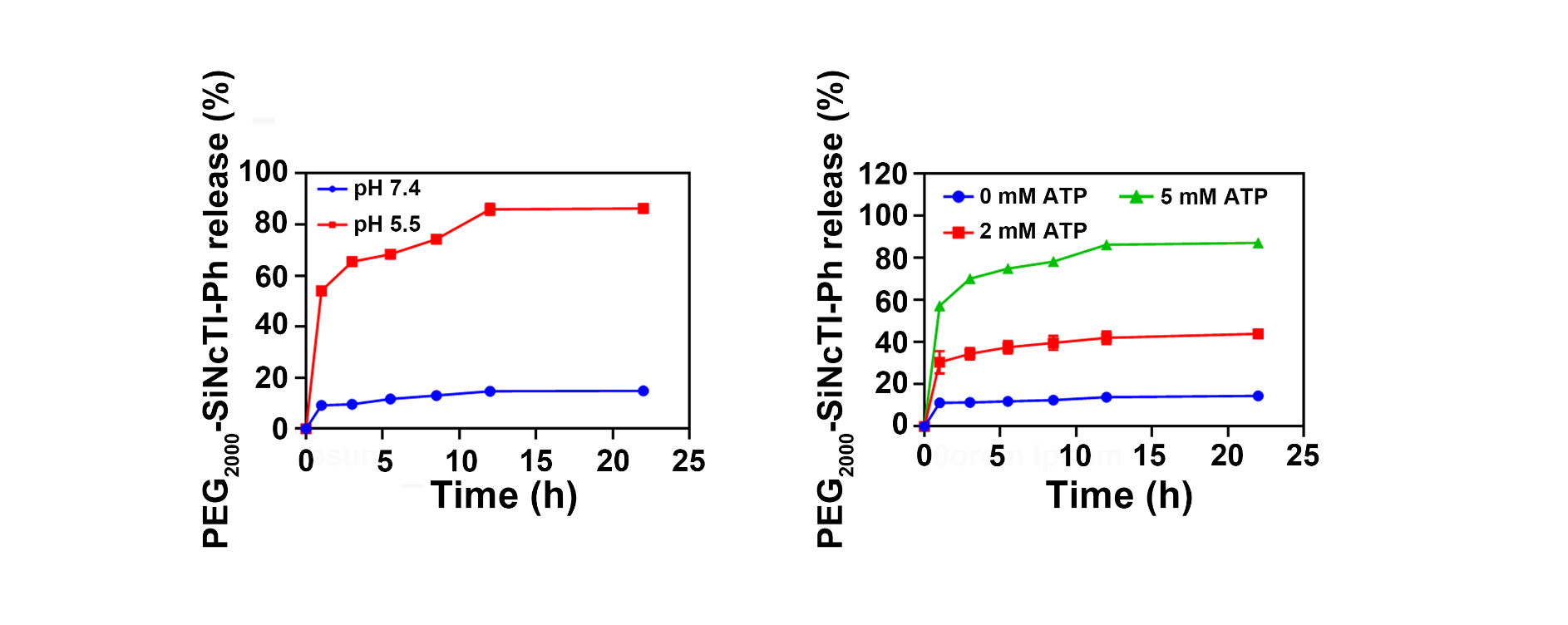


**Figure S18.** PEG_2000_-SiNcTI-Ph release from PEG_2000_-SiNcTI-Ph/CpG-ZIF-8@CM in PBS at different pH (left) or with different concentrations of ATP (right). Data are presented as mean ± S.D. (n = 3 independent experiments per group).


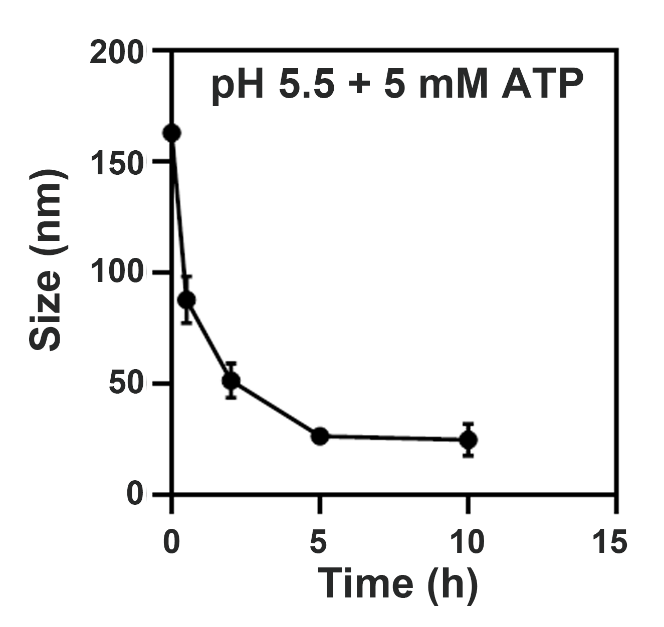


**Figure S19.** Changes of particle size of PEG_2000_-SiNcTI-Ph/CpG-ZIF-8@CM over time incubated in PBS with low pH (5.5) plus high concentrations of ATP (5 mM).


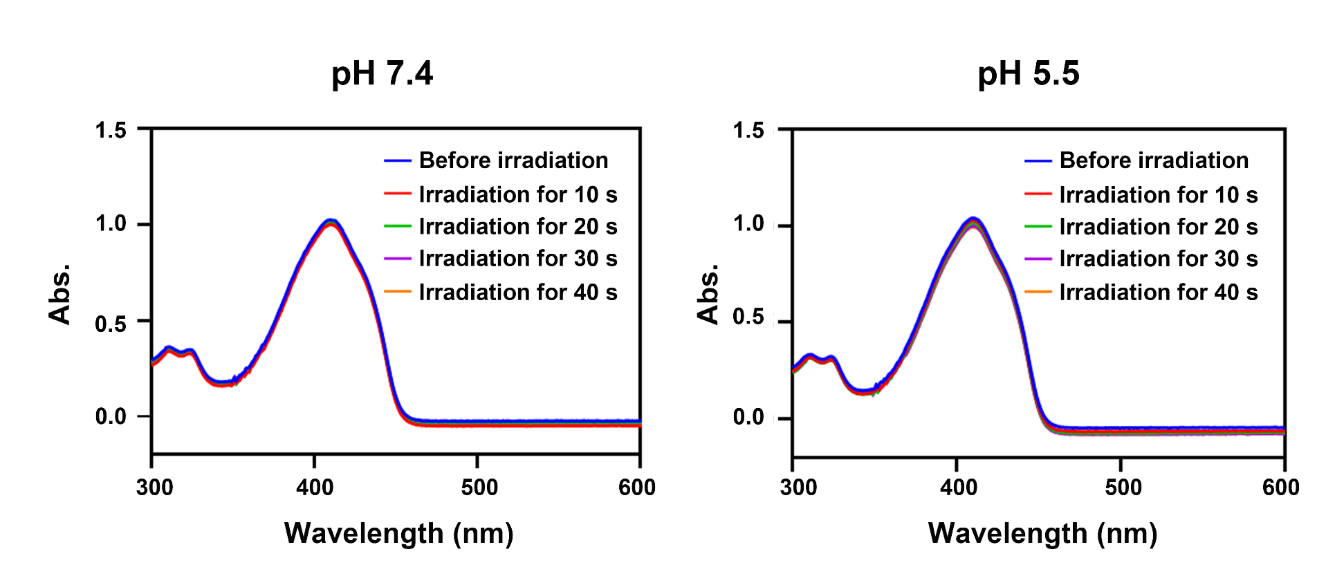


**Figure S20.** ROS generation ability of PEG_2000_-SiNcTI-Ph/CpG-ZIF-8@CM incubated in PBS of different pH for 0 h, which was detected by DPBF at 410 nm.


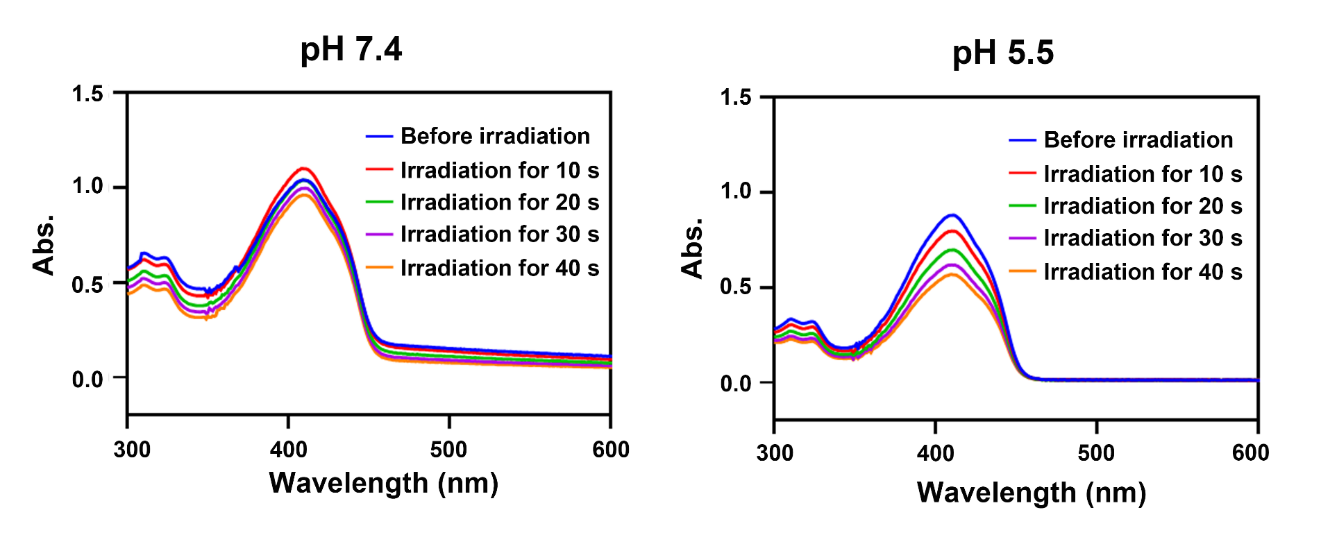


**Figure S21.** ROS generation ability of PEG_2000_-SiNcTI-Ph/CpG-ZIF-8@CM incubated in PBS of different pH for 4 h, which was detected by DPBF at 410 nm.


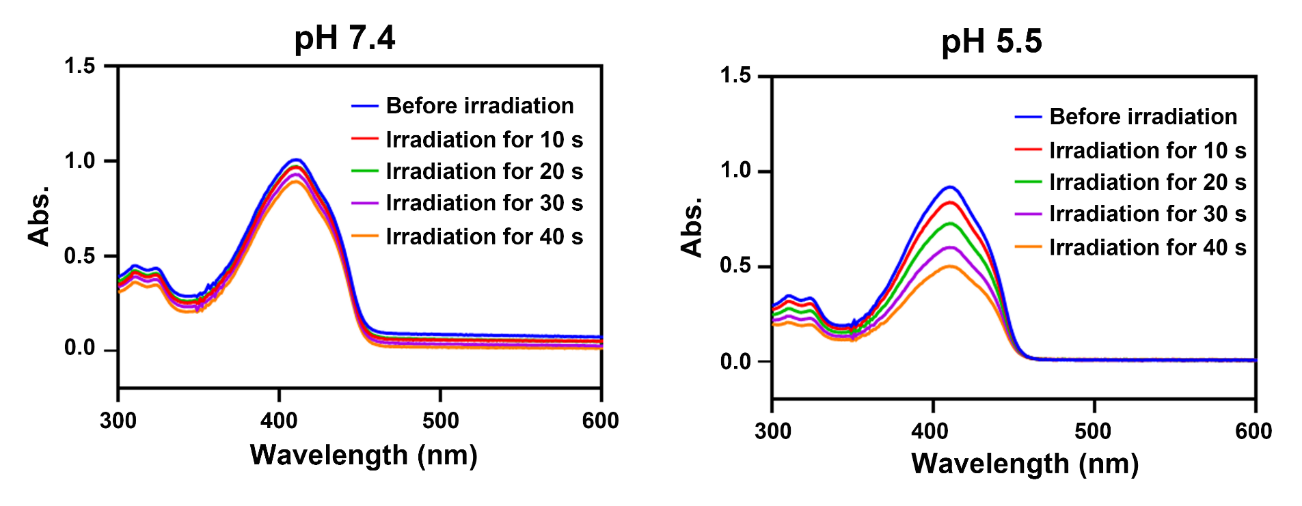


**Figure S22.** ROS generation ability of PEG_2000_-SiNcTI-Ph/CpG-ZIF-8@CM incubated in PBS of different pH for 8 h, which was detected by DPBF at 410 nm.


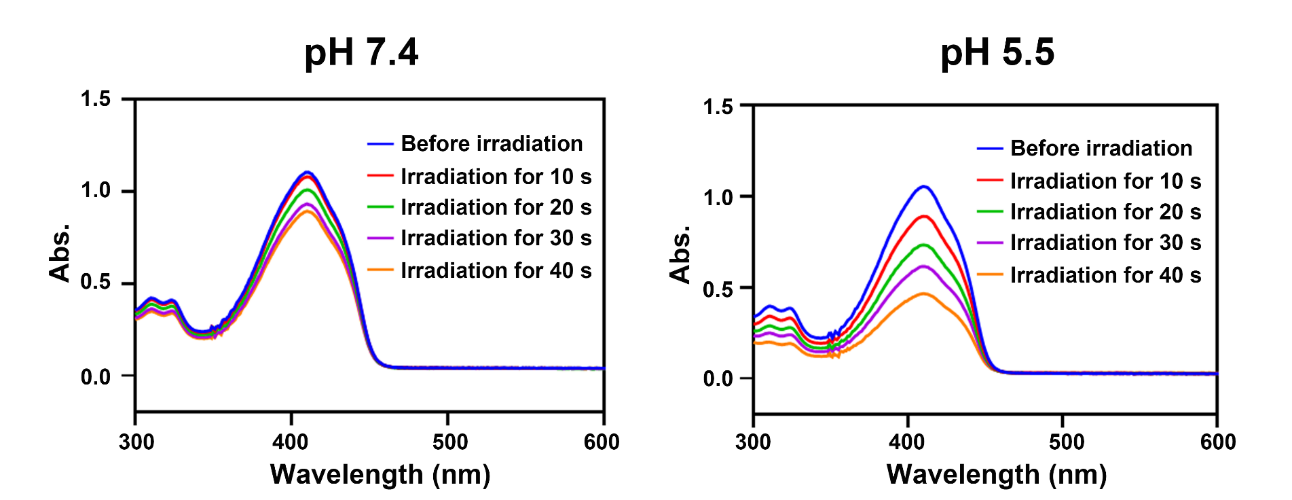


**Figure S23.** ROS generation ability of PEG_2000_-SiNcTI-Ph/CpG-ZIF-8@CM incubated in PBS of different pH for 12 h, which was detected by DPBF at 410 nm.


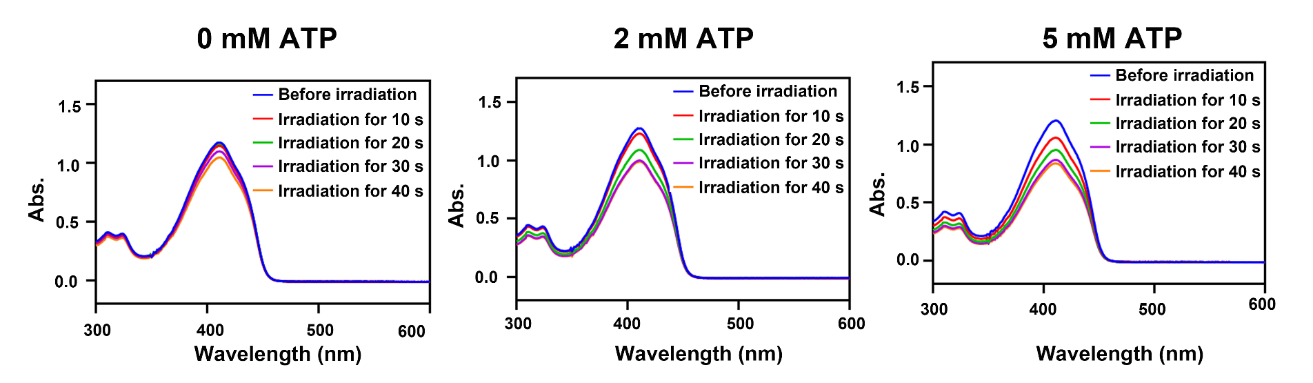


**Figure S24.** ROS generation ability of PEG_2000_-SiNcTI-Ph/CpG-ZIF-8@CM incubated in PBS with different concentrations of ATP for 4 h, which was detected by DPBF at 410 nm.


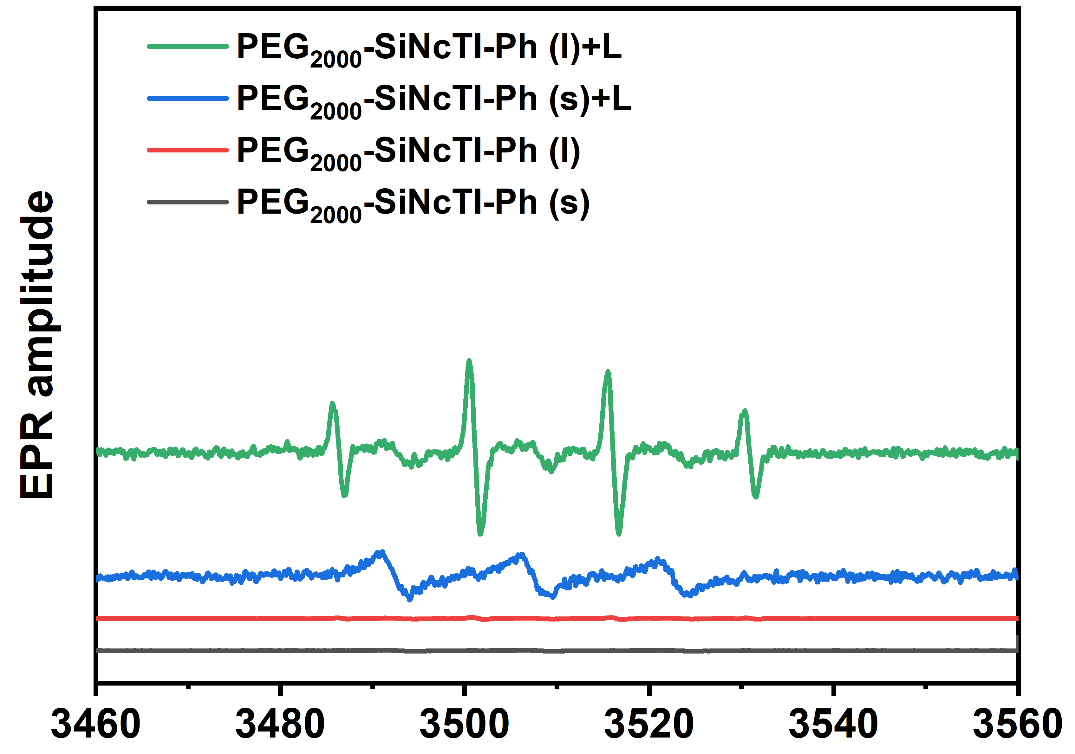


**Figure S25.** EPR signals of PEG_2000_-SiNcTI-Ph in solid (aggregated state) and liquid (water solution, dispersed state) conditions with or without laser irradiation (808 nm, 0.5 W/cm^2^, 3 min). "s" and "l" represent "solid" and "liquid", respectively.


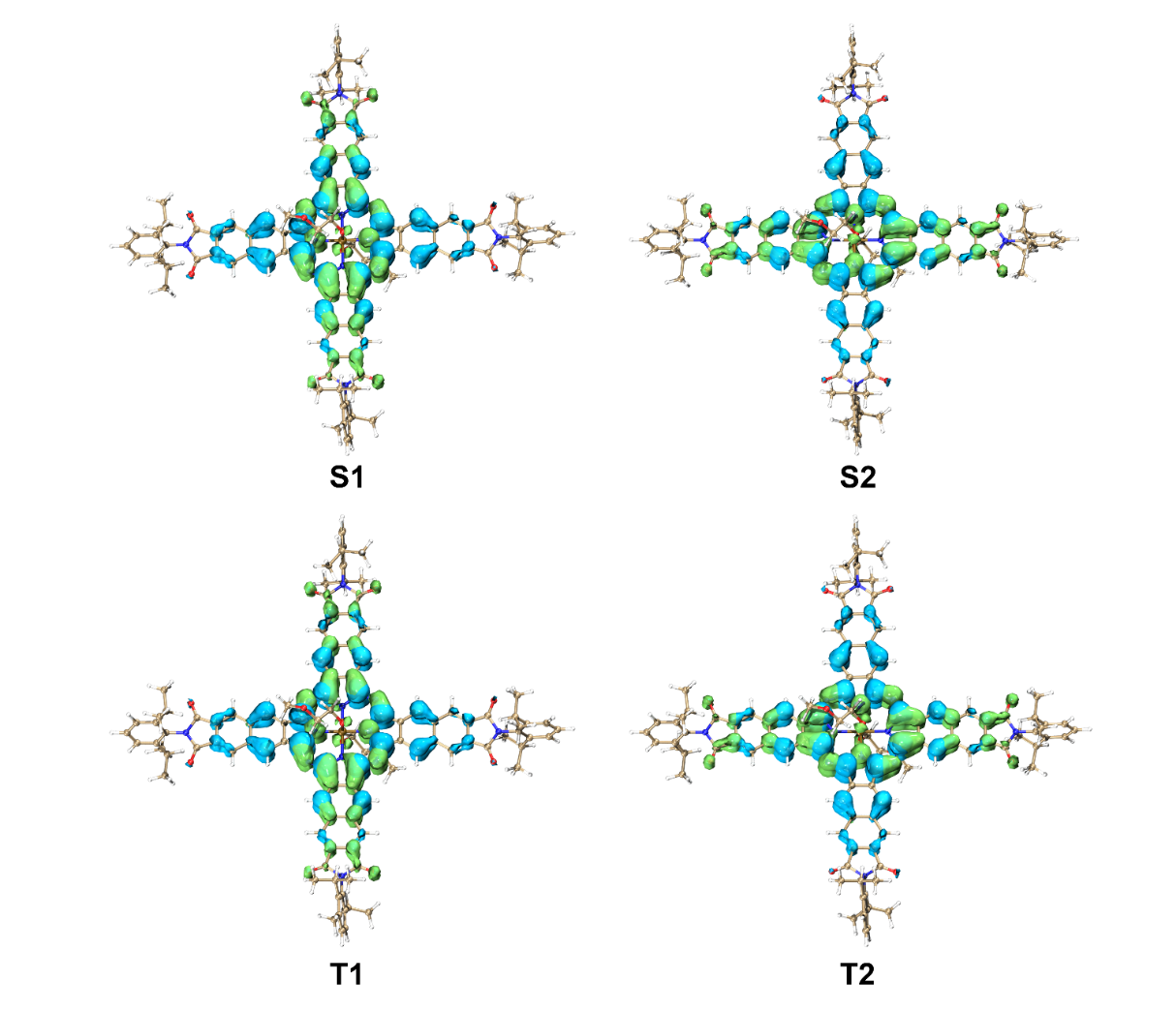


**Figure S26.** The hole-electron analysis for the lowest singlet (S1 and S2) and triplet (T1 and T2) excited states based on the monomer model.


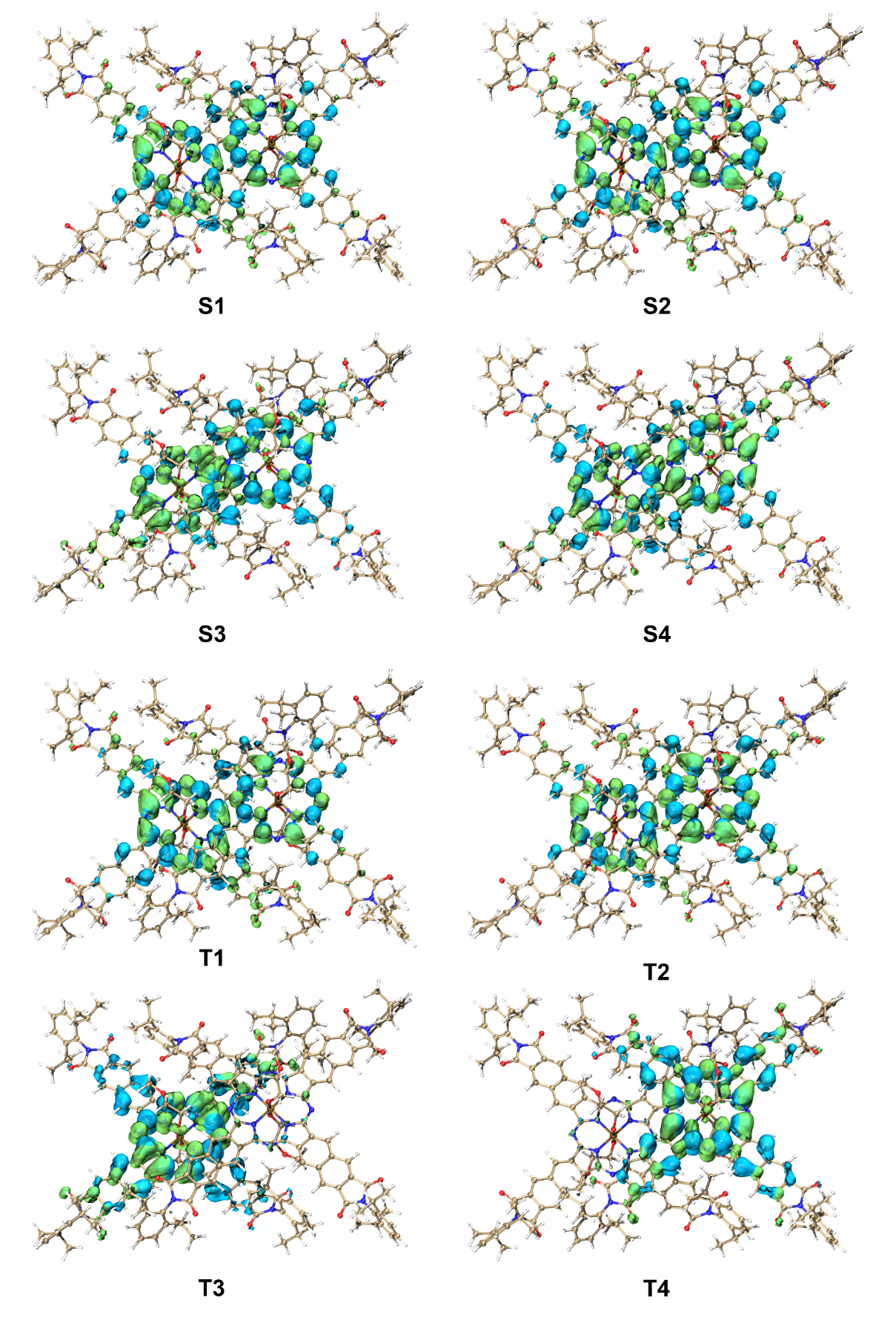


**Figure S27.** The hole-electron analysis for the lowest singlet (S1-S4) and triplet (T1-T4) excited states based on the dimer model.


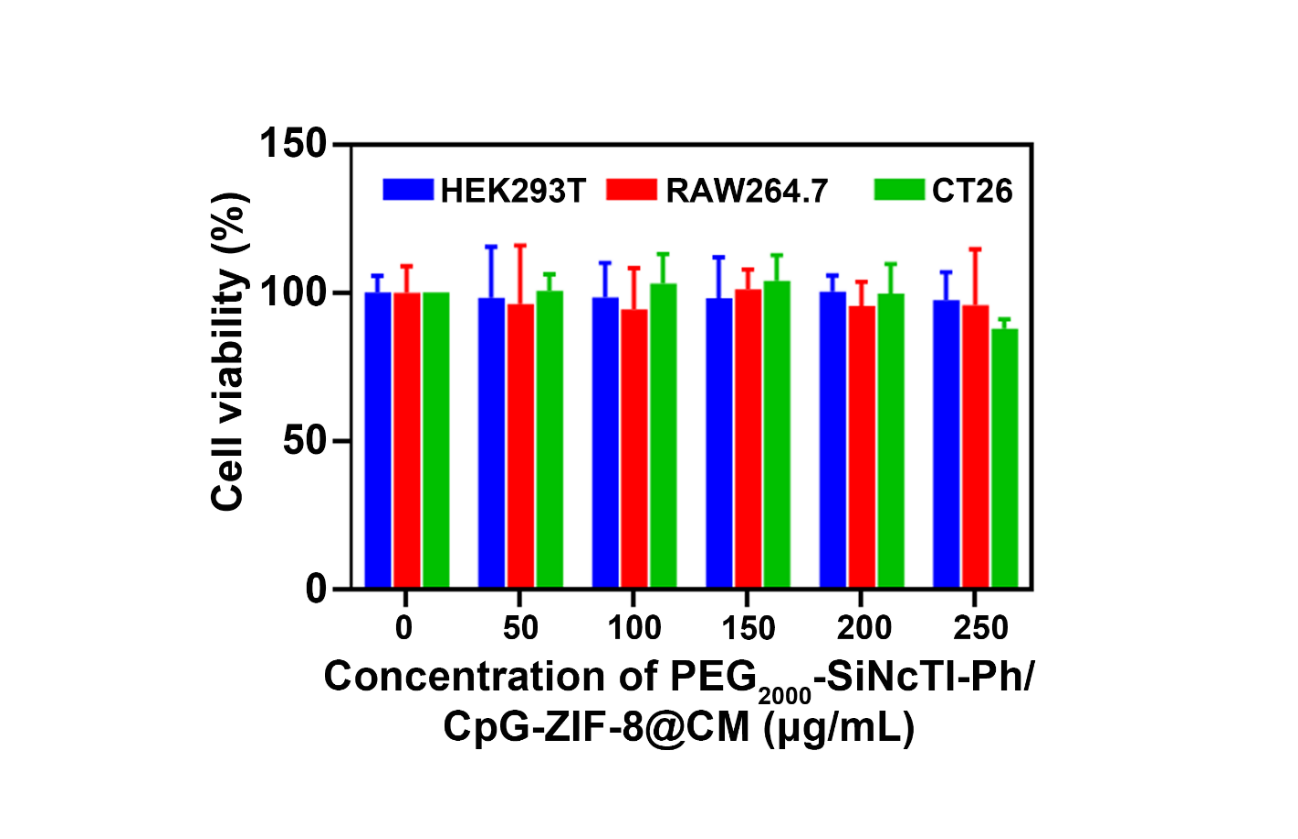


**Figure S28.** HEK293T, RAW264.7, and CT26 cell viabilities after incubation with different concentrations of PEG_2000_-SiNcTI-Ph/CpG-ZIF-8@CM. Data are presented as mean ± S.D. (n = 3 biologically independent experiments per group).


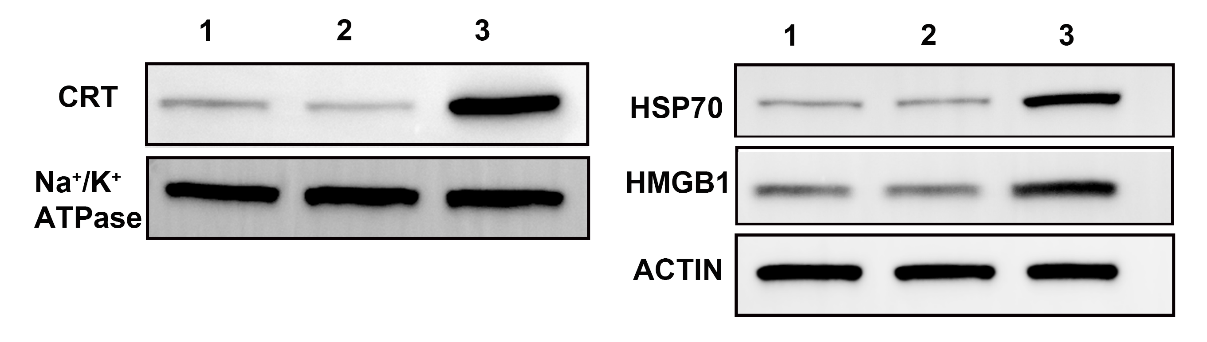


**Figure S29.** WB analysis of CRT presented in cell membrane and HMGB-1/HSP70 in the supernatant after different treatments (1: PBS, 2: PEG_2000_-SiNcTI-Ph/CpG-ZIF-8@CM, 3: PEG_2000_-SiNcTI-Ph/CpG-ZIF-8@CM+L (plus 808 nm laser irradiation)).


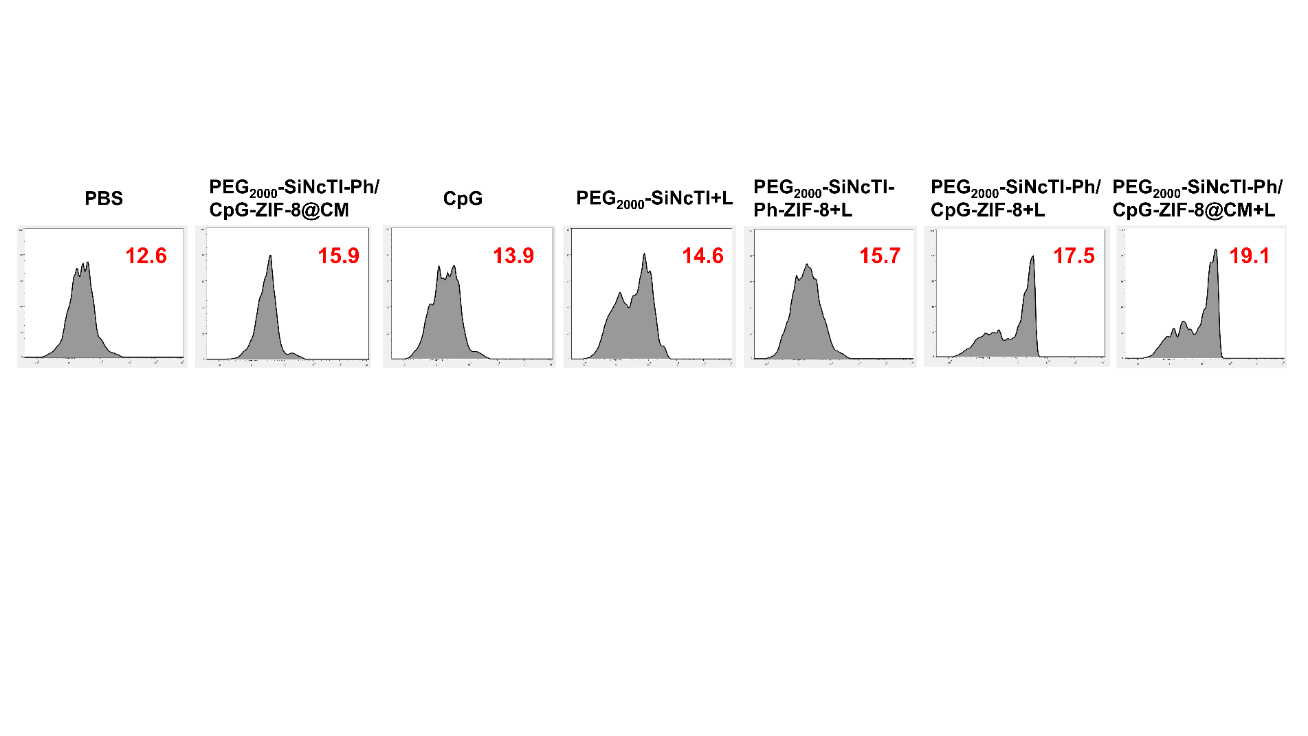


**Figure S30.** Flow cytometry analysis of CD8^+^ T cell percentage in splenic immune cells co-incubated with the supernatants of CT26 cells after different treatments.


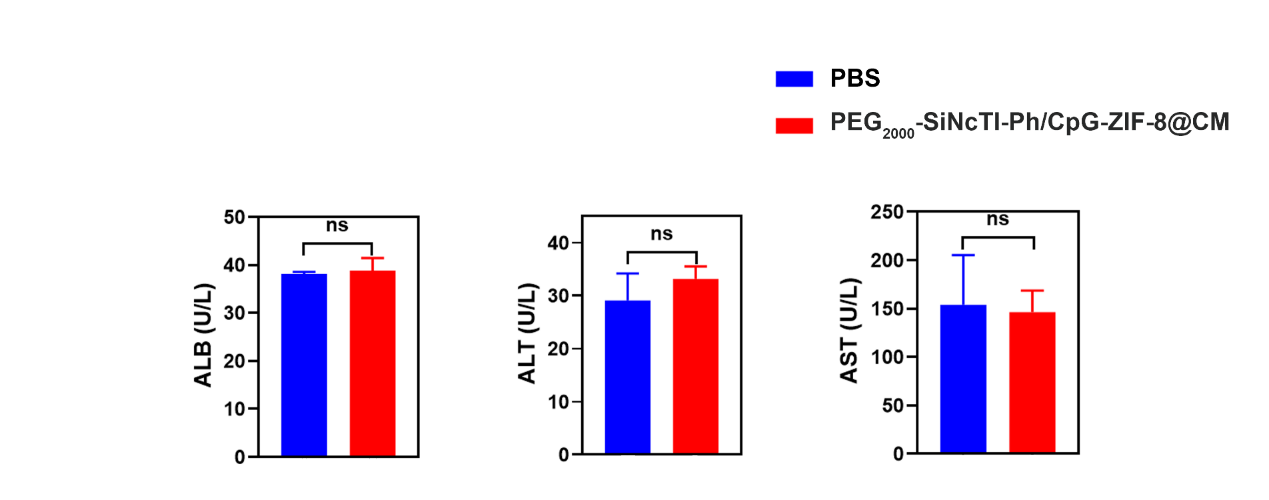


**Figure S31.** Biochemical indicators analysis of liver injury from the mice blood with or without PEG_2000_-SiNcTI-Ph/CpG-ZIF-8@CM administration. Statistical analysis was performed using the two-sided Student's t-test. "ns" represents no significant difference. Data are presented as mean ± S.D. (n = 3 biologically independent experiments per group).


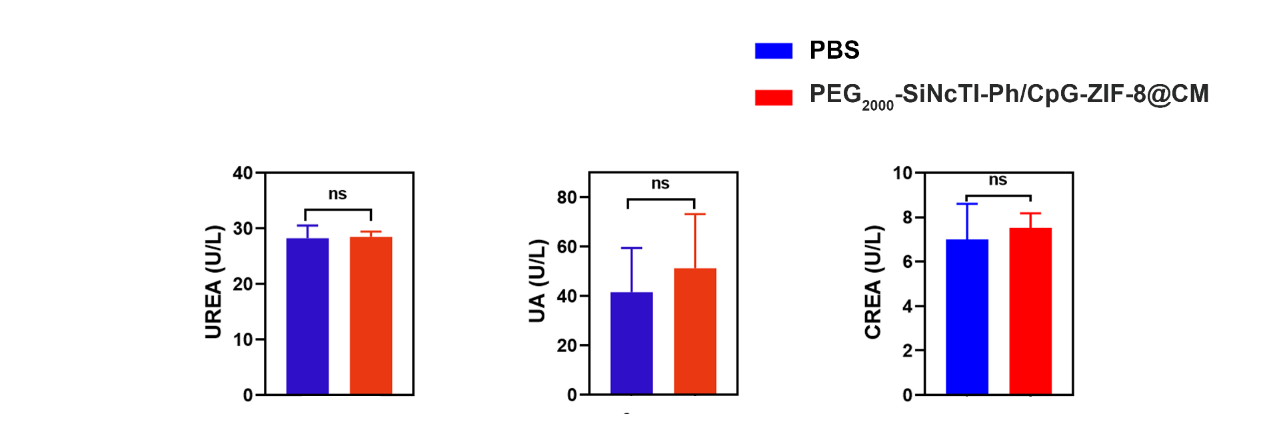


**Figure S32.** Biochemical indicators analysis of kidney injury from the mice blood with or without PEG_2000_-SiNcTI-Ph/CpG-ZIF-8@CM administration. Statistical analysis was performed using the two-sided Student's t-test. "ns" represents no significant difference. Data are presented as mean ± S.D. (n = 3 biologically independent experiments per group).


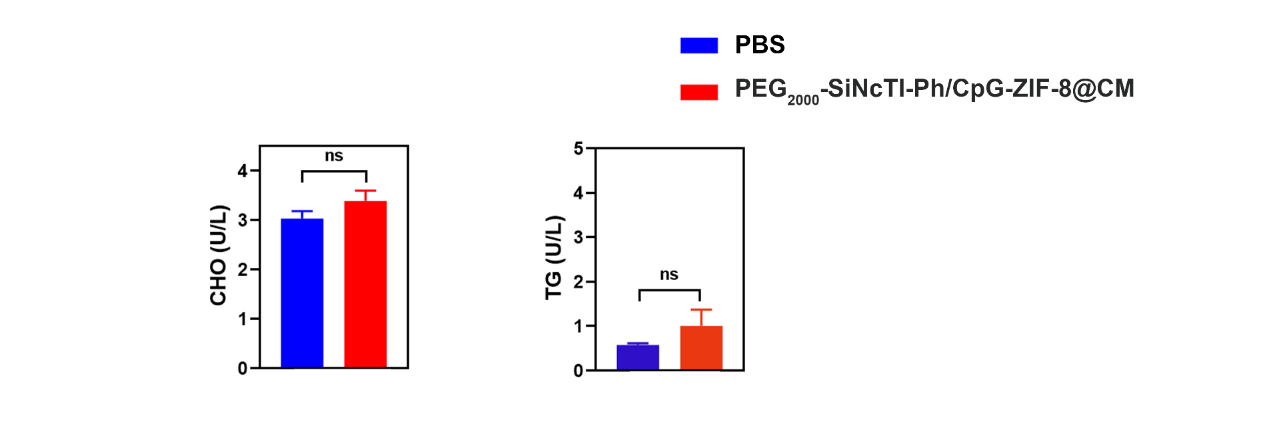


**Figure S33.** Biochemical indicators analysis of lipids from the mice blood with or without PEG_2000_-SiNcTI-Ph/CpG-ZIF-8@CM administration. Statistical analysis was performed using the two-sided Student's t-test. "ns" represents no significant difference. Data are presented as mean ± S.D. (n = 3 biologically independent experiments per group).


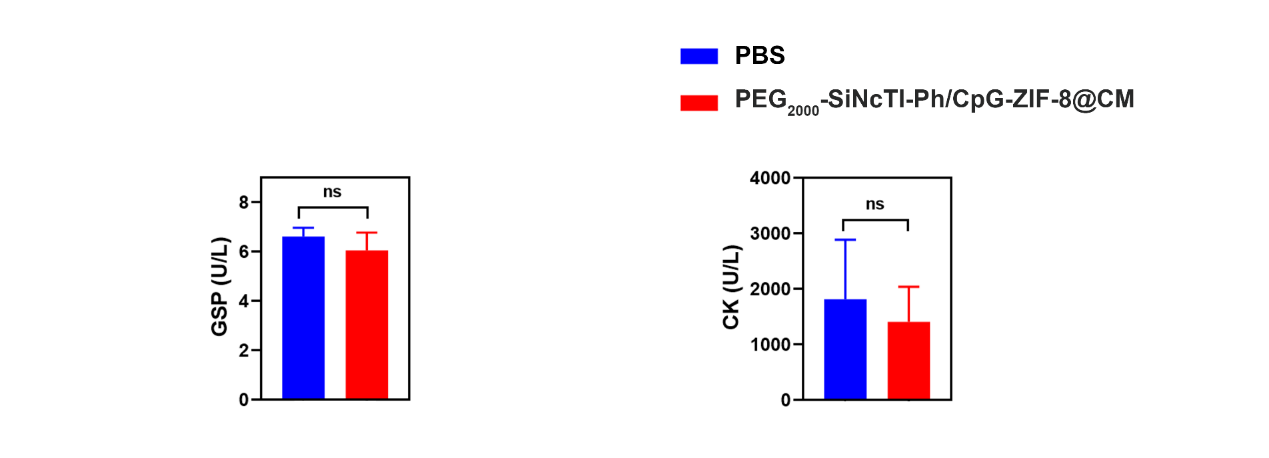


**Figure S34.** Biochemical indicators analysis of blood glucose (left) and cardiac enzyme profiles (right) from the mice blood with or without PEG_2000_-SiNcTI-Ph/CpG-ZIF-8@CM administration. Statistical analysis was performed using the two-sided Student's t-test. "ns" represents no significant difference. Data are presented as mean ± S.D. (n = 3 biologically independent experiments per group).


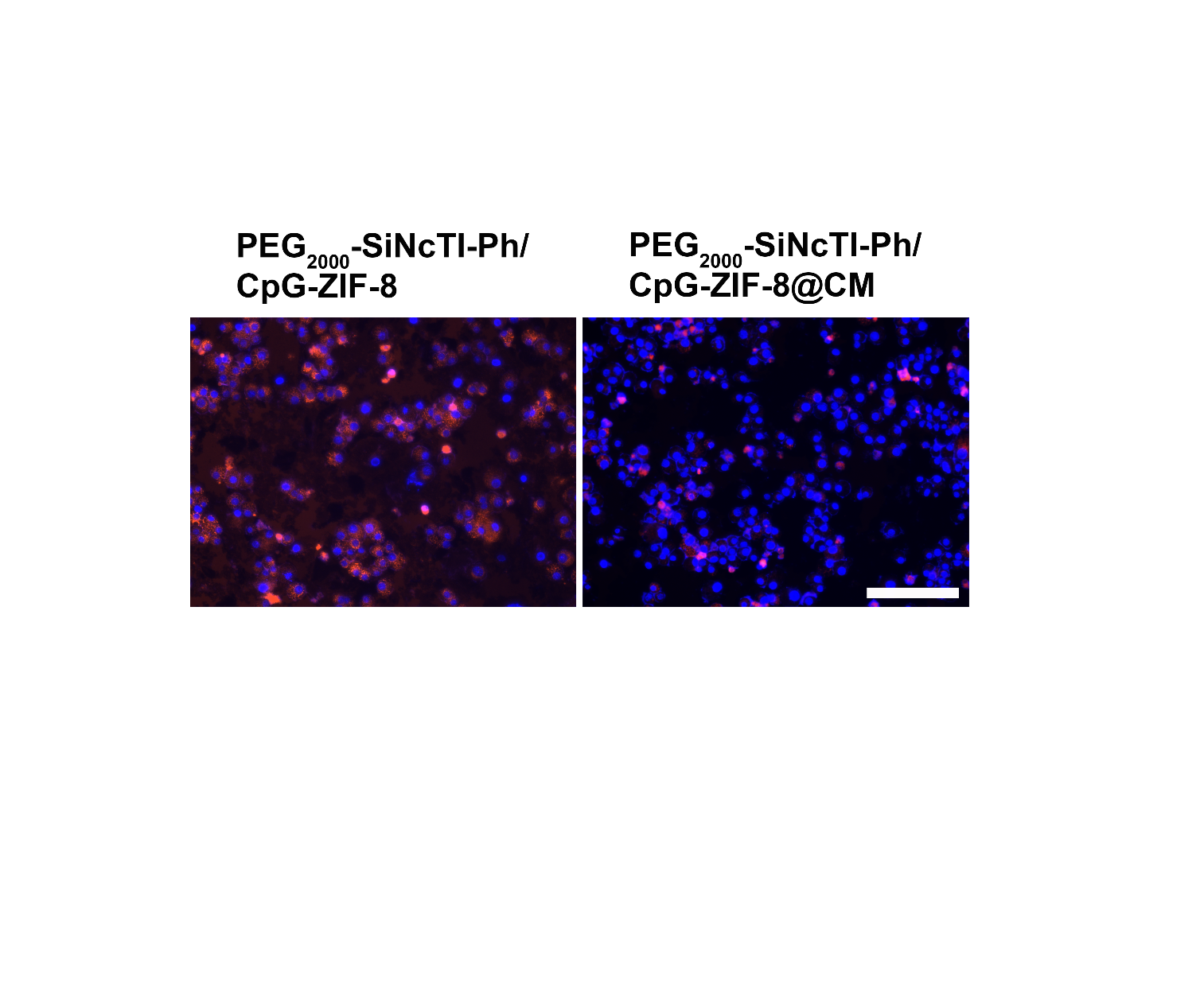


**Figure S35.** Fluorescence images of RAW264.7 cells after co-incubation with PEG_2000_-SiNcTI-Ph/CpG-ZIF-8 or PEG_2000_-SiNcTI-Ph/CpG-ZIF-8@CM. Scale bar, 150 μm.


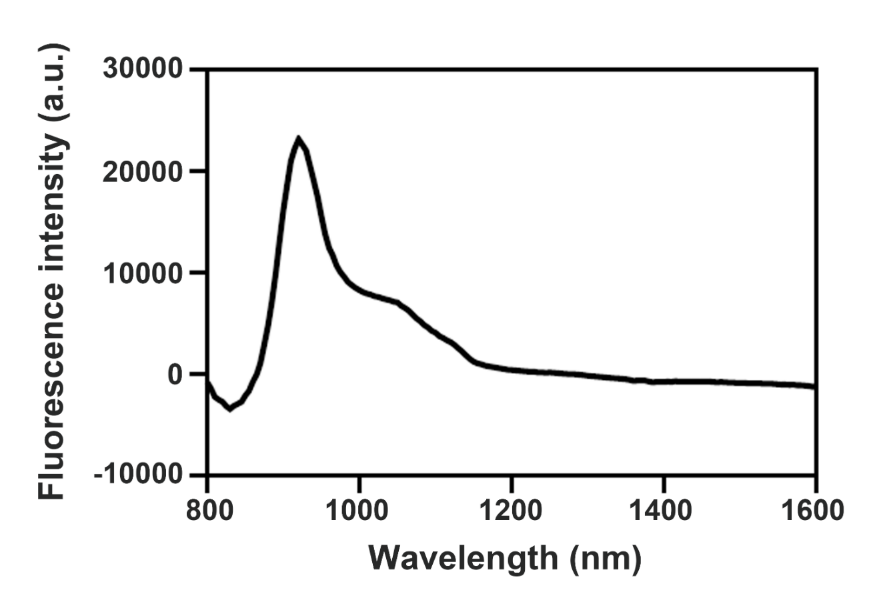


**Figure S36.** Fluorescence spectrum of PEG_2000_-SiNcTI-Ph.


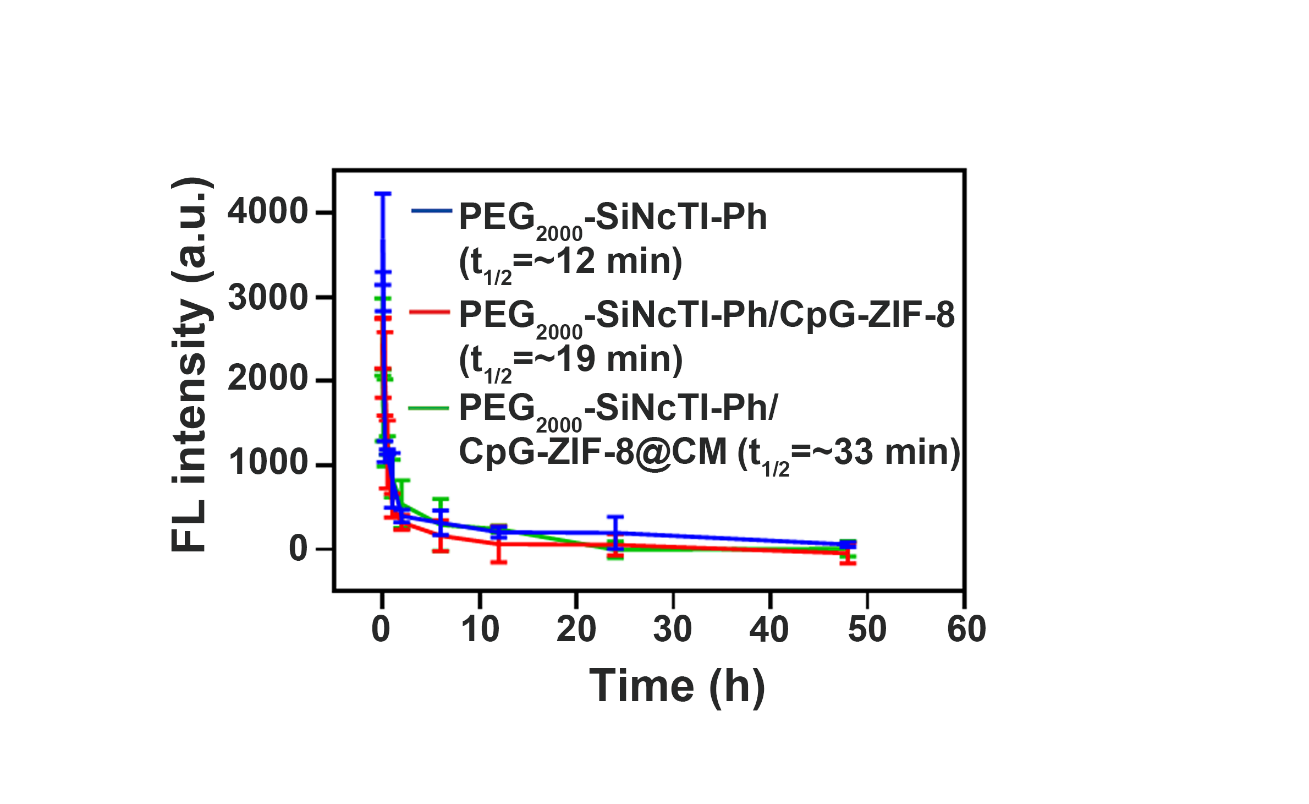


**Figure S37.** *In vivo* blood circulation of different materials. Mouse blood specimens were collected for fluorescence imaging and spectral analysis at different time points after intravenous injection of PEG_2000_-SiNcTI-Ph, PEG_2000_-SiNcTI-Ph/CpG-ZIF-8, or PEG_2000_-SiNcTI-Ph/CpG-ZIF-8@CM (PEG_2000_-SiNcTI-Ph equivalent dose: 2.5 mg/kg body weight). Data are presented as mean ± S.D. (n = 3 biologically independent experiments per group).


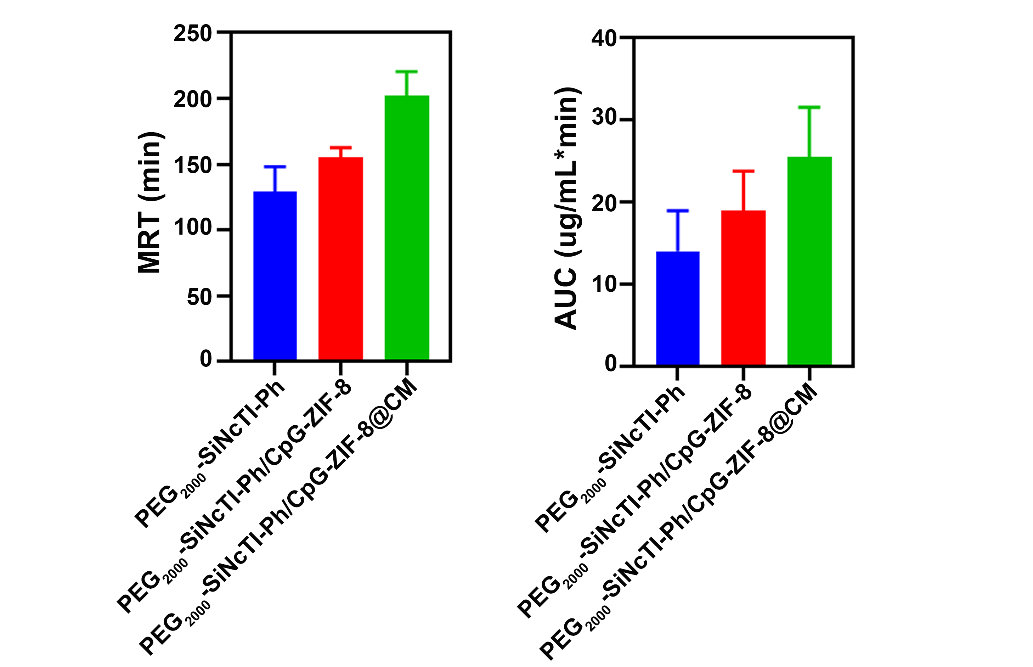


**Figure S38.** Pharmacokinetic parameters of different materials.


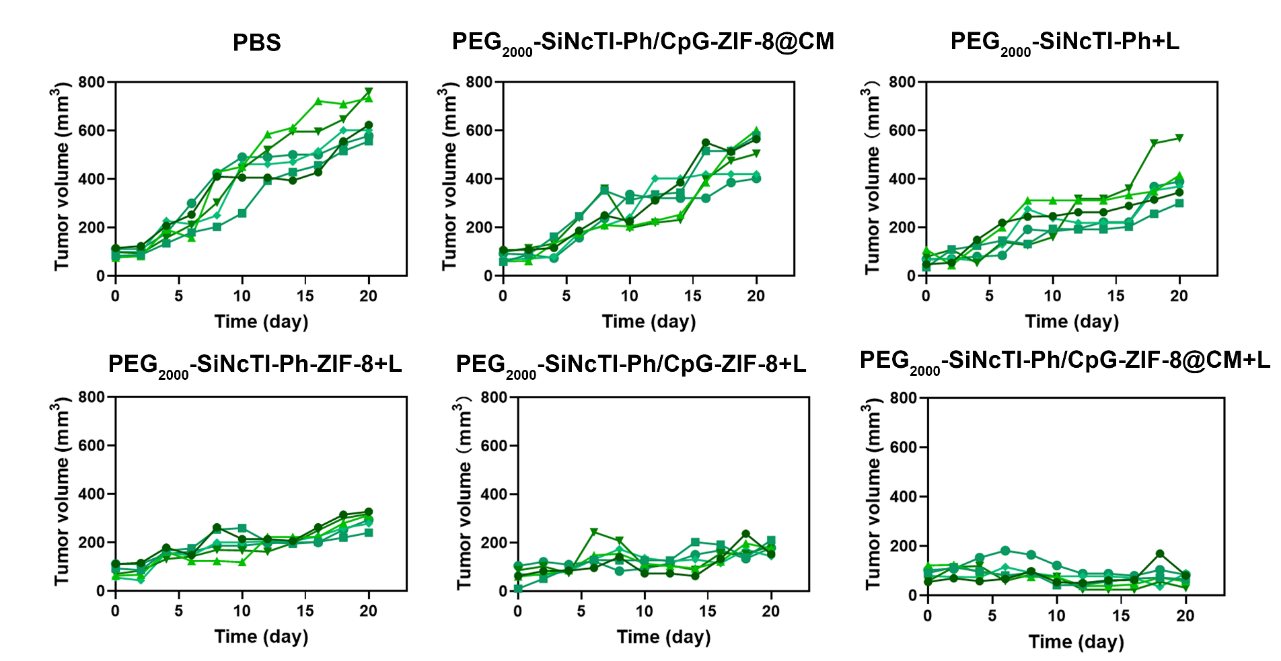


**Figure S39.** Primary tumor growth curves of CT26 tumor-bearing mice (n = 6).


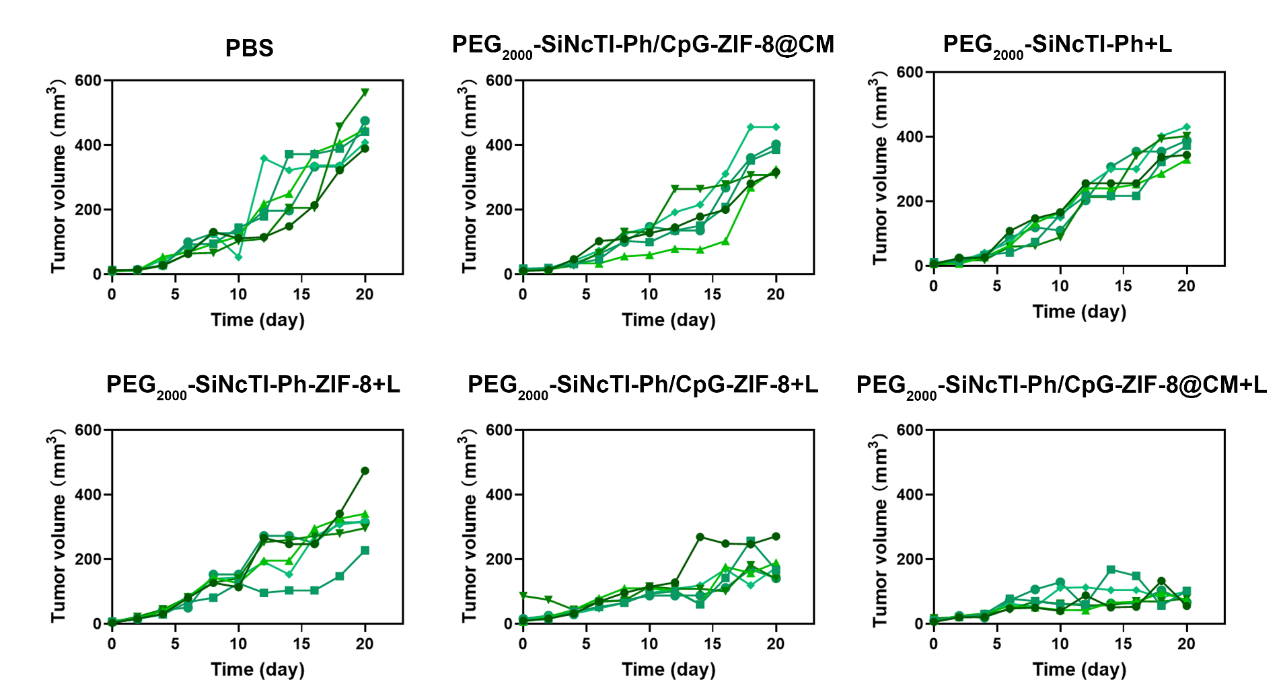


**Figure S40.** Distant tumor growth curves of CT26 tumor-bearing mice (n = 6).


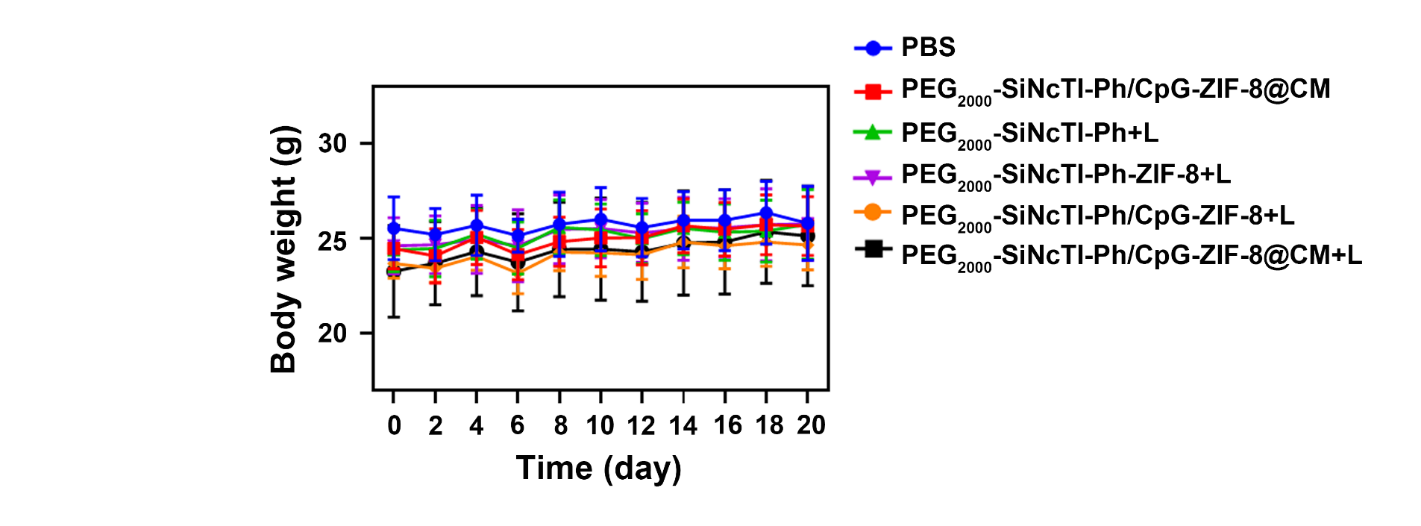


**Figure S41.** Mouse body weight variation over time in different groups. Data are presented as mean ± S.D. (n = 6 biologically independent experiments per group).


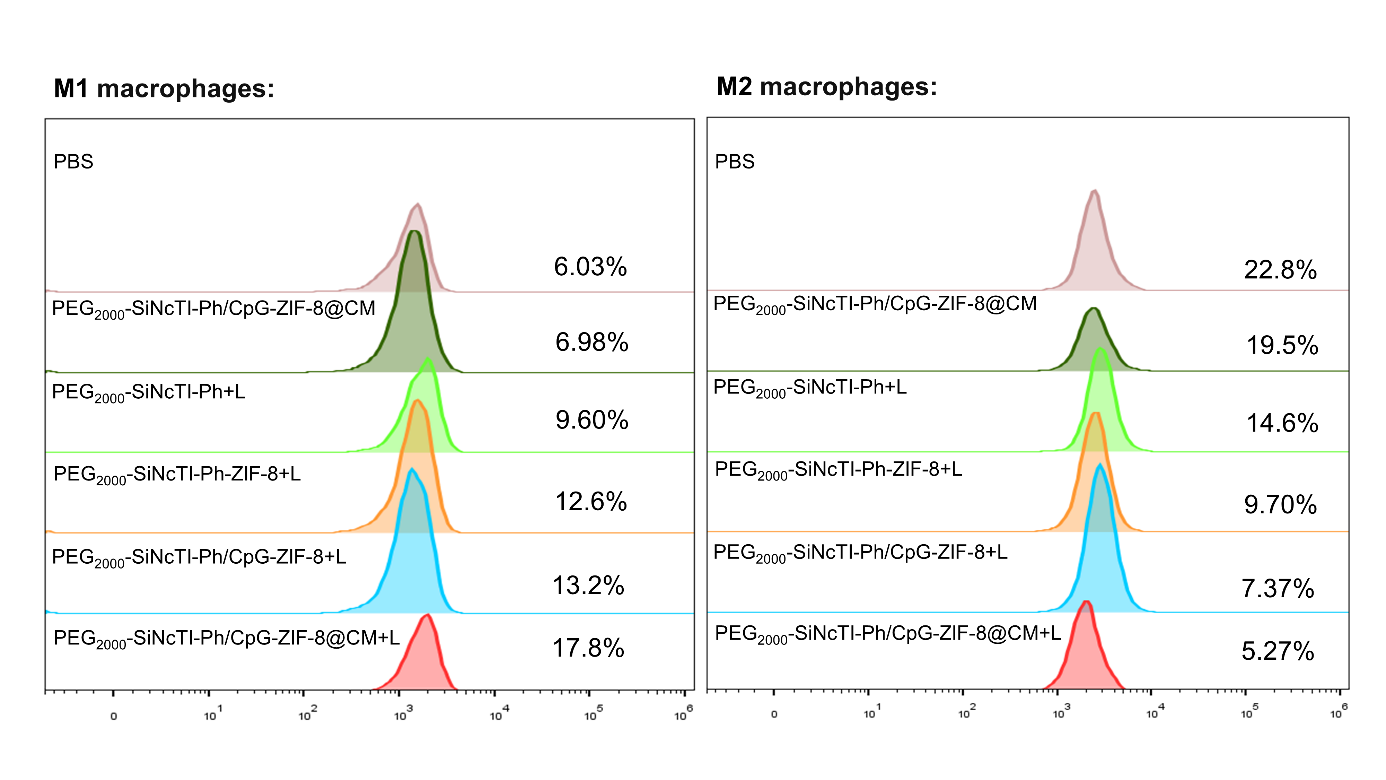
**Figure S42.** Flow cytometric analysis of macrophage phenotypes in solid CT26 tumors after different treatments.


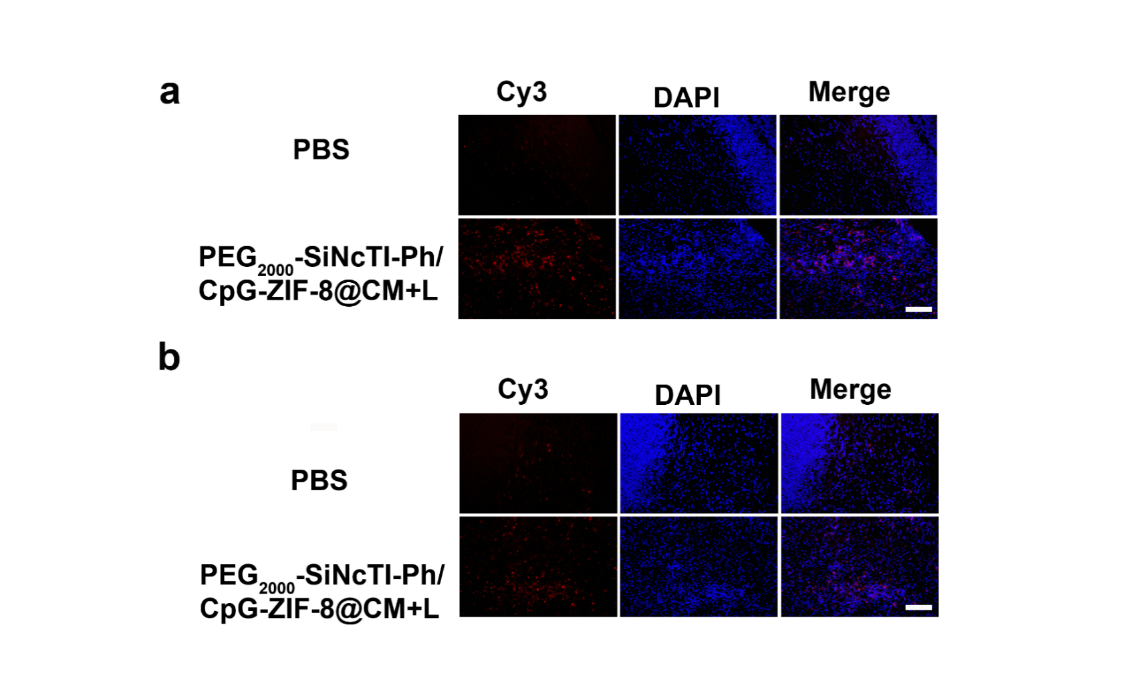


**Figure S43.** Immunofluorescence analysis of intratumoral infiltration of (**a**) CD4^+^ and (**b**) CD8^+^ T cells in CT26 tumor-bearing mice. Scale bar, 100 μm. Red: Both of CD4 and CD8 were labeled by Cy3. Blue: Cell nuclei were stained by DAPI.

**Table S1.** Calculated vertical S_0_-S_n_ excitation energies (E_0n_), oscillator strength (f_0n_), and electronic configurations determined at various PCM (water)-TD-DFT/6-31G(d) level of theory.

| Methods | | B3LYP/6-31G (d) | | | ωB97XD/6-31G (d) | | M062X/6-31G (d) | | _EXP._ |
| --- | --- | --- | --- | --- | --- | --- | --- | --- | --- |
| Models | | **E_0n_ (λ_0n_)**  **eV (nm)** | **f_0n_** | **Electronic Configurations** | **E_0n_ (λ_0n_)**  **eV (nm)** | **f_0n_** | **E_0n_ (λ_0n_)**  **eV (nm)** | **f_0n_** | **(nm)** |
| Monomer | S1 | **1.63 (766)** | 1.00 | **HOMO → LUMO 97%** | **1.63 (759)** | 1.06 | **1.71 (724)** | 1.10 | ~800 |
|  | S2 | **1.64 (755)** | 1.00 | **HOMO → LUMO+1 97%** | **1.65 (750)** | 1.07 | **1.73 (717)** | 1.11 | - |
|  | T1 | **0.91 (1365)** | 0.00 | **HOMO → LUMO 100%** | **Wavefunction** **Unstable** | - | **0.76 (1634)** | 0.00 |  |
|  | T2 | **0.92 (1346)** | 0.00 | **HOMO → LUMO+1 99%** |  |  | **0.78 (1593)** | 0.00 |  |
| Dimer | S1 | **1.41 (878)** | 0.14 | **HOMO → LUMO 81%**  **HOMO-1 → LUMO+2 14%** |  | | | | |
|  | S2 | **1.43 (867)** | 0.01 | **HOMO-1 → LUMO 70%**  **HOMO-1 → LUMO+2 20%** |  |  |  |  |  |
|  | S3 | **1.46 (848)** | 0.05 | **HOMO → LUMO+1 68%**  **HOMO-1 → LUMO+3 16%** |  |  |  |  |  |
|  | S4 | **1.48 (837)** | 0.00 | **HOMO-1 → LUMO+1 50%**  **HOMO → LUMO+3 31%** |  |  |  |  |  |
|  | T1 | **0.85 (1461)** | 0.00 | **HOMO → LUMO 60%**  **HOMO-1 → LUMO+2 32%** |  |  |  |  |  |
|  | T2 | **0.86 (1444)** | 0.00 | **HOMO-1 → LUMO 50%**  **HOMO → LUMO+2 41%** |  |  |  |  |  |
|  | T3 | **0.92 (1355)** | 0.00 | **HOMO → LUMO+1 42%**  **HOMO-1 → LUMO+3 29%** |  |  |  |  |  |
|  | T4 | **0.93 (1336)** | 0.00 | **HOMO → LUMO+3 37%**  **HOMO-1 → LUMO+1 32%** |  |  |  |  |  |
